# Characterization of the morphology and complete mitochondrial genomes of *Eupteryx minusula* and *Eupteryx gracilirama* (Hemiptera: Cicadellidae: Typhlocybinae) from Karst area, Southwest China

**DOI:** 10.7717/peerj.12501

**Published:** 2021-11-24

**Authors:** Zhouwei Yuan, Kangning Xiong, Ni Zhang, Can Li, Yuehua Song

**Affiliations:** 1 Guizhou Normal University, School of Karst Science, Guiyang, Guizhou, The People’s Republic of China; 2 Guiyang University, Guizhou Provincial Key Laboratory for Rare Animal and Economic Insect of the Mountainous Region, Guiyang, Guizhou, The People’s Republic of China

**Keywords:** Typhlocybinae, Mitochondrial genome, Morphology, Characterization

## Abstract

**Background:**

The hemipteran insect family Cicadellidae (leafhoppers) includes >2,600 valid genera and >22,000 valid species worldwide, including >2,000 species in China. Typhlocybinae, second largest subfamilies of Cicadellidae, is widely distributed in the six major zoogeographic regions of the world, including >4,000 species worldwide and >1,000 species in China. Previously, morphological analysis are often effective to the way of taxonomy, but it did not combine with molecular biology. Therefore, morphology and mitochondrial genomes (mitogenomes) of two leafhopper species, *Eupteryx* (*Eupteryx*) *minuscula* Lindberg, 1929 and *Eupteryx* (*Stacla*) *gracilirama* Hou, Zhang & Huang, 2016 were studied and analyzed. This study analyzed the morphological and molecular characteristics of the two leafhoppers, and showed whether the results of the two identifications were consistent.

**Methods:**

Based on the method of comparison, mitogenomes and morphology were analyzed to prove the relationship between the two leafhoppers.

**Results:**

Although two focal species are classified in two different subgenera of the same genus, they still share many morphological features, such as the moderately produced crown fore margin; the milky yellow apical part of scutellum; the pronotum, basal triangles of scutellum, and forewing are dark with several colorless patches on the surface; the light yellow face, without any spots or stripes, and so on. The circular mitogenomes are 16,944 bp long in *E. minuscula* (GenBank: MN910279) and 17,173 bp long in *E. gracilirama* (GenBank: MT594485). All of the protein-coding genes are starting with ATN, except for some in mitogenome, which has a single T or TAN as a stop codon. All tRNAs have the typical cloverleaf-shaped structure except for *trnS1* (AGN) (*E. minuscula*) which has a reduced DHU arm. Moreover, these two mitogenomes have *trnR* with an unpaired base in the acceptor stem. The phylogenetic relationships between *E. minuscula* and *E. gracilirama* in respect to related lineages were reconstructed using Maximum likelihood and Maximum parsimony analyses.

**Discussion:**

The result showed that the tribe Typhlocybini is a sister to the tribes Erythroneurini and Empoascini, and five genera, *Bolanusoides*, *Typhlocyba*, *Eupteryx*, *Zyginella* and *Limassolla* are forming a single clade. *E. minuscula* and *E. gracilirama* are clustered together, supporting the monophyly of the genus *Eupteryx*. The above conclusions are consistent with the traditional classification of the subfamily.

## Introduction

The hemipteran insect family Cicadellidae (leafhoppers) includes >2,600 valid genera and >22,000 valid species worldwide, including >2,000 species in China (Oman, Knight & Nielson, 1990; Dietrich, 2005). Typhlocybinae, second largest subfamilies of Cicadellidae, is widely distributed in the six major zoogeographic regions of the world, including >4,000 species worldwide and >1,000 species in China (Dmitriev, 2003-onward). The leafhopper genus *Eupteryx* was established by Curtis (1829) with *Cicada atropunctata* Goeze, 1778 as its type species by subsequent designation by Curtis (1837). It includes two subgenera *Eupteryx* and *Stacla*, and comprises 123 known species (Dmitriev, 2003-onward). All leafhoppers are phytophagous, feeding exclusively on plants; they are abundant in forests and grasslands (Morris, 1971; Hamilton, 1996). Some species are important agricultural pests and vectors of plant pathogens (Miao & Han, 2008; Zhu, He & Wei, 2012).

The insect mitogenomes are closed, double-stranded DNA molecule, which measures approximately 14–19 kb in size and contains 13 protein-coding genes (PCGs), 22 transfer RNA (tRNA) genes, two ribosomal RNA (rRNA) genes, and a noncoding region (Boore, 1999; Wang et al., 2015). Although, mitogenomes are being more and more extensively used for evolutionary, phylogenetic, and population genetic studies (Nardi et al., 2003; Cook, Yue & Akam, 2005; Cameron, 2014; Li et al., 2017), the mitogenomes of leafhoppers, especially those Typhlocybini species are remaining rarely explored, particularly that of the phylogenetic relationship of Typhlocybini and Zyginellini. This two tribes were traditionally distinguished based on differences in hind wing venation. Recently the latter was considered to be a junior synonym of the former (Dietrich, 2013). The relationships and phylogenetic status of these two tribes have not yet been tested by previous analyses of DNA sequence data and morphological characteristics (Dietrich, 2004; Dietrich et al., 2017). So, these should be used to further investigate their phylogenetic relationships.

In this study, we present and analyze the complete mitogenomes and the external morphology of two leafhopper species, *E. minuscula* Lindberg, 1929 and *E. gracilirama* Hou, Zhang & Huang, 2016, including the gene order, nucleotide composition, codon usage, gene overlaps, intergenic regions, non-coding regions, the body habitus, and the characters of male/female genitalia. Using newly obtained sequences, along with previously published mitogenomes of Typhlocybinae (Table 1), the phylogenetic relationships were reconstructed based on the concatenated nucleotide sequences of 13 PCGs.

**Table 1:** Taxonomic information and GenBank accession numbers for the species used in this study.

Subfamily/Tribe	Species	Accession no.	Reference
Typhlocybinae/Empoascini	*Ghauriana sinensis* (Qin & Zhang, 2011)	MN699874	Shi, Yu & Yang (2020)
Typhlocybinae/Empoascini	*Empoasca vitis* (Göthe, 1875)	KJ815009	Zhou et al. (2016)
Typhlocybinae/Empoascini	*Empoasca flavescens*	MK211224	Luo et al. (2019)
Typhlocybinae/Empoascini	*Empoasca* sp.15062818	MK251093	Song, Zhang & Zhao (2019)
Typhlocybinae/Empoascini	*Kybos pura* (Stål, 1858)	MK251088	Song, Zhang & Zhao (2019)
Typhlocybinae/Empoascini	*Empoasca onukii* (Matsuda, 1952)	MK251089	Song, Zhang & Zhao (2019)
Typhlocybinae/Empoascini	*Empoasca* sp. C2591422	MK251111	Song, Zhang & Zhao (2019)
Typhlocybinae/Empoascini	*Empoasca* sp.	MK251099	Song, Zhang & Zhao (2019)
Typhlocybinae/Zyginellini	*Limassolla lingchuanensis* (Chou & Zhang, 1985)	MN605256	Yuan et al. (2020)
Typhlocybinae/Zyginellini	*Zyginella minuta* (Yang, 1965)	MT488436	Han et al. (2020)
Typhlocybinae/Typhocybini	*Bolanusoides shaanxiensis* (Huang & Zhang, 2005)	MN661136	Yan et al. (2019)
Typhlocybinae/Typhocybini	*Typhlocyba* sp. EMHAU 15062510	KY039138	Song, Cai & Li (2017)
Typhlocybinae/Typhocybini	*Eupteryx* (*Stacla*) *gracilirama* (Hou, Zhang & Huang, 2016)	MT594485	This study
Typhlocybinae/Typhocybini	*Eupteryx* (*Eupteryx*) *minuscula* (Lindberg, 1929)	MN910279	Yang et al. (2020)
Typhlocybinae/Erythroneurini	*Eratoneura flexibilis* (Knull, 1949)	MK251094	Song, Zhang & Zhao (2019)
Typhlocybinae/Erythroneurini	*Mitjaevia protuberanta* (Song, Li & Xiong, 2011)	MN627216	Yuan, Li & Song (2020)
Typhlocybinae/Erythroneurini	*Empoascanara dwalata* (Dworakowska, 1971)	MT350235	Chen, Li & Song (2021)
Typhlocybinae/Erythroneurini	*Empoascanara sipra* (Dworakowska, 1980)	MN604278	Tan et al. (2020)
Typhlocybinae/Erythroneurini	*Illinigina* sp. EMHAU 15062817	KY039129	Song, Cai & Li (2017)
Typhlocybinae/Erythroneurini	*Erythroneura vitifex* (Fitch, 1856)	MK251100	Song, Zhang & Zhao (2019)
Typhlocybinae/Erythroneurini	*Zygina ordinaria* (Ribaut, 1936)	MK251104	Song, Zhang & Zhao (2019)
Outgroup	*Macrosteles quadrilineatus* (Forbes, 1885)	KY645960	Mao, Yang & Bennett (2017)
Outgroup	*Japananus hyalinus* (Osborn, 1900)	KY129954	Du et al. (2017)
Outgroup	*Yanocephalus yanonis* (Matsumura, 1902)	KY039113	Song, Cai & Li (2017)

## Materials & Methods

### Ethics statement

Collections were not done from any national park or another protected area of land or sea, or on any private land, hence no permission was required. No specific permissions were required for any of the collection localities/activities, as the collections were done in or around the demonstration area of Karst rocky desertification Control, Guizhou Province, China. The field studies did not involve any endangered or protected species. Specimens were collected by sweeping a net from different locations in this area and processed by a series of steps such as sorting, cleaning, and mounting.

### Morphology

Complete specimens of leafhoppers were taken out of the 95% ethanol solution. The excess of ethanol was sucked dry with qualitative filter paper, and the posture was adjusted. Leafhoppers were photographed in dorsal, lateral, and ventral views using KEYENCE VHX-5000C. Male/female specimens were selected under a stereoscope, the entire abdomen of the specimens was removed and soaked in 10% NaOH solution or 10% KOH solution for 15–20 h. After that, the abdomen was rinsed with clean water, drained with qualitative filter paper of the excess water and transferred to a clean glass dripping with glycerine. An Olympus SZX16 dissecting microscope was used for specimen study and Olympus BX53 stereoscopic microscope was used for taking pictures of the dissected male genitalia and female genitalia. The rest of the specimen with a label was stored in 95% ethyl alcohol and placed in a refrigerator at −20 °C.

## Mitochondrial Genomics

### Samples and DNA extraction

All of the studied specimens were collected in Huajiang Town, Guizhou Province, China, from May to October, 2019. Species were identified following Hou, Zhang & Huang (2016) and Lindberg (1929). The specimens were preserved in ethanol and stored in the insect specimen storage room of Guizhou Normal University with the accession numbers GZNU-ELS-2019011 and GZNU-ELS-2019012. The leafhoppers were washed twice by vortexing in absolute ethanol and dried at room temperature before DNA extraction. Genomic DNA was extracted by using a tissue rapid Extraction Kit (VWI). The insects were incubated at 56 °C for 6 h to lyse completely and total genomic DNA was eluted in 50 µl double-distill water (ddH_2_O), while the remaining steps were conducted following the manufacturer’s protocol. Genomic DNA was stored at −20 °C.

### Primer design, PCR amplification and sequencing

Primers were designed based on conserved regions sequences and used to amplify short fragments of mitogenome sequences using PCR methodology. The PCR reaction was performed using the LA Taq polymerase and following conditions: pre-denaturation 94 °C for 2 min, then 35 cycles of denaturation at 94 °C for 30 s, annealing at 55 °C for 30 s, and extension at 72 °C for 1 min/kb, followed by the final extension at 72 °C for 10 min. The final concentration of the forward and reverse primers was 0.2–1.0 mM, and that of MgCl_2_ was 2.0 mM. The PCR products were sequenced directly, or if needed first cloned into a pMD18-T vector (Takara, JAP) and then sequenced, by the dideoxy nucleotide procedure, using an ABI 3730 automatic sequencer (Sanger sequencing) and the same set of primers.

### Assembly, gene annotation and sequence analysis

After quality-proofing of the obtained fragments, the complete mitogenome sequences were assembled manually using DNA star v7.1 software (Burland, 2000). Mitogenomes were annotated roughly following the procedure described before (Zhang et al., 2018). The assembled mitogenomes were compared with those of other shibing species and identified by BLAST searches in the NCBI database (Altschul et al., 1997) to ensure that the sequences were correct. The locations of PCGs were annotated using ORF Finder *via* NCBI with the selection of invertebrate mitogenome sequence. Abnormal start codons and stop codons were determined based on comparisons with other invertebrate insects. The position and secondary structure of 22 tRNA genes were annotated using tRNAscan-SE version 1.21 (Lowe & Eddy, 1997) and ARWEN version 1.2 (Laslett & Canbäck, 2008). The *16sRNA* genes and *12sRNA* genes were determined based on the locations of adjacent tRNA genes and by comparisons with other hemipterans. Circular mitogenome maps were drawn using CG View (http://stothard.afns.ualberta.ca/cgview_server/) (Grant & Stothard, 2008) and Photoshop CC 2020. Codon usage, composition, skewness of wases, and relative synonymous codon usage (RSCU) were analyzed using PhyloSuite v1.1.15 (Zhang et al., 2018). Strand asymmetry was calculated using the formulae: AT skew = (A − T)/(A + T); and GC skew = (G − C)/(G + C) (Perna & Kocher, 1995).

### Phylogenetic analysis

So far, about 30 complete or near-complete mitogenomes of Typhlocybinae have been published in the National Center for Biotechnology Information (NCBI) database. Mitogenomes of 21 Typhlocybinae species were selected, containing eight species from Empoascini, seven species from Erthroneurini, four species from Typhlocybini, two species from Zyginellini. *Macrosteles quadrilineatus*, *Japananus hyalinus* and *Yanocephalus yanonis* were regarded as an outgroup (Table 1). PCGs genes were aligned using the MAFFT v 7.313 (Wong, Suchard & Huelsenbeck, 2008). The phylogenetic tree was reconstructed based on Maximum Likelihood (ML) and Maximum Parsimony (MP) in phyML (Guindon et al., 2005) and Mega X (Sudhir et al., 2018). For the ML analysis, the best model selection was done with Model test 3.7 (Posada & Crandall, 1998) and the GTR+G+I model was optimal for analysis with nucleotide alignment.

## Results

### Taxonomy based on morphology

Head in dorsal view bluntly produced, somewhat narrower than pronotum. Male pygofer lobe with macrosetae at cephalo-ventral angle, with anal tube processes, and with or without various sclerotized process along posterior margin. Subgenital plate with single macrosetae basally. Style with long blade-like apex. Connective Y-shaped, with robust stem. Aedeagal shaft usually with paired processes; preatrium well developed. So far, the genus *Eupteryx* consists of two subgenera *Eupteryx* and *Stacla*. The former pygofer side with various processes at hind margin; basal single macroseta on subgenital plate shorter and the latter pygofer side without any process at hind margin; basal single macroseta on subgenital plate longer. Both of them can be distinguished with the characteristics mentioned above.

### *Eupteryx (Eupteryx) minuscula* Lindberg, 1929

*Eupteryx minuscula* Lindberg, 1929: 12; Vilbaste, 1968: 90; Dworakowska, 1970: 363; Anufriev, 1978:154; Dworakowska, 1982: 170.

*Eupteryx ussuriensis* Vilbaste, 1966: 63

**Figure 1:**
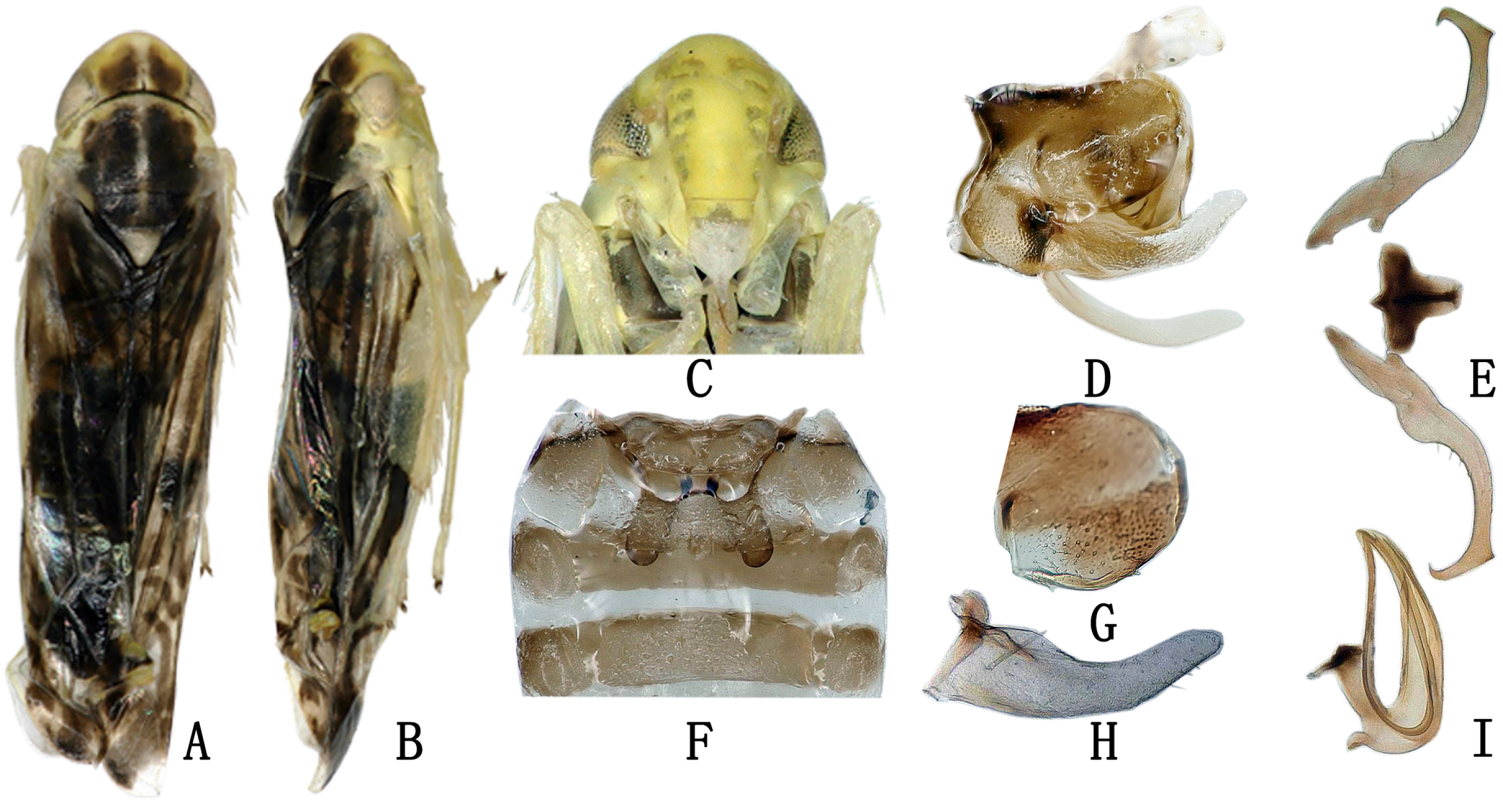
The morphological characteristics of male *E*. (*Eupteryx*) *minuscula*. (A) Dorsal view. (B) lateral view. (C) Face. (D) Pygofer, lateral view. (E) Connective & styles. (F) Abdominal apodemes. (G) Pygofer lobe. (H) Subgenital plate. (I) Aedeagus, later view.

**Description.** Body dull yellow. Crown roundly produced medially, somewhat narrower than pronotum. Vertex brownish yellow and eyes grey. Pronotum with symmetrical large brown patches medially. Mesonotum with brown lateral triangles and milky yellow apex (Fig. 1A). Forewing dark brown, with several irregular whitish patches on surface, color pattern very similar to another species, *Eupteryx* (*Stacla*) *gracilirama* (Fig. 1B). Abdomen light brown in dorsal view and yellow in ventral view, except for somewhat darker apical segment (Fig. 1C). Abdominal apodemes extending to 4th sternite (Fig. 1F).

**Male genitalia.** Pygofer lobe rounded, with several small rigid setae apically; ventral appendage sclerotized, tapering towards apex and curved upward (Figs. 1D, 1G). Subgenital plate long and slender, expanded at base and with row of microsetae at upper margin apically (Fig. 1H). Style with foot-like apex (Fig. 1E), with heel little larger than in *Eupteryx* (*Stacla*) *gracilirama*. Aedeagal shaft with pair of long and slender apical processes (Fig. 1I), processed directed basad, reaching the base of shaft, recurving dorsad at middle to reach the tip of aedeagus, forming U or V-shaped curve in lateral view. Connective with broad and short arms and distinct central lobe (Fig. 1E).

**Figure 2:**
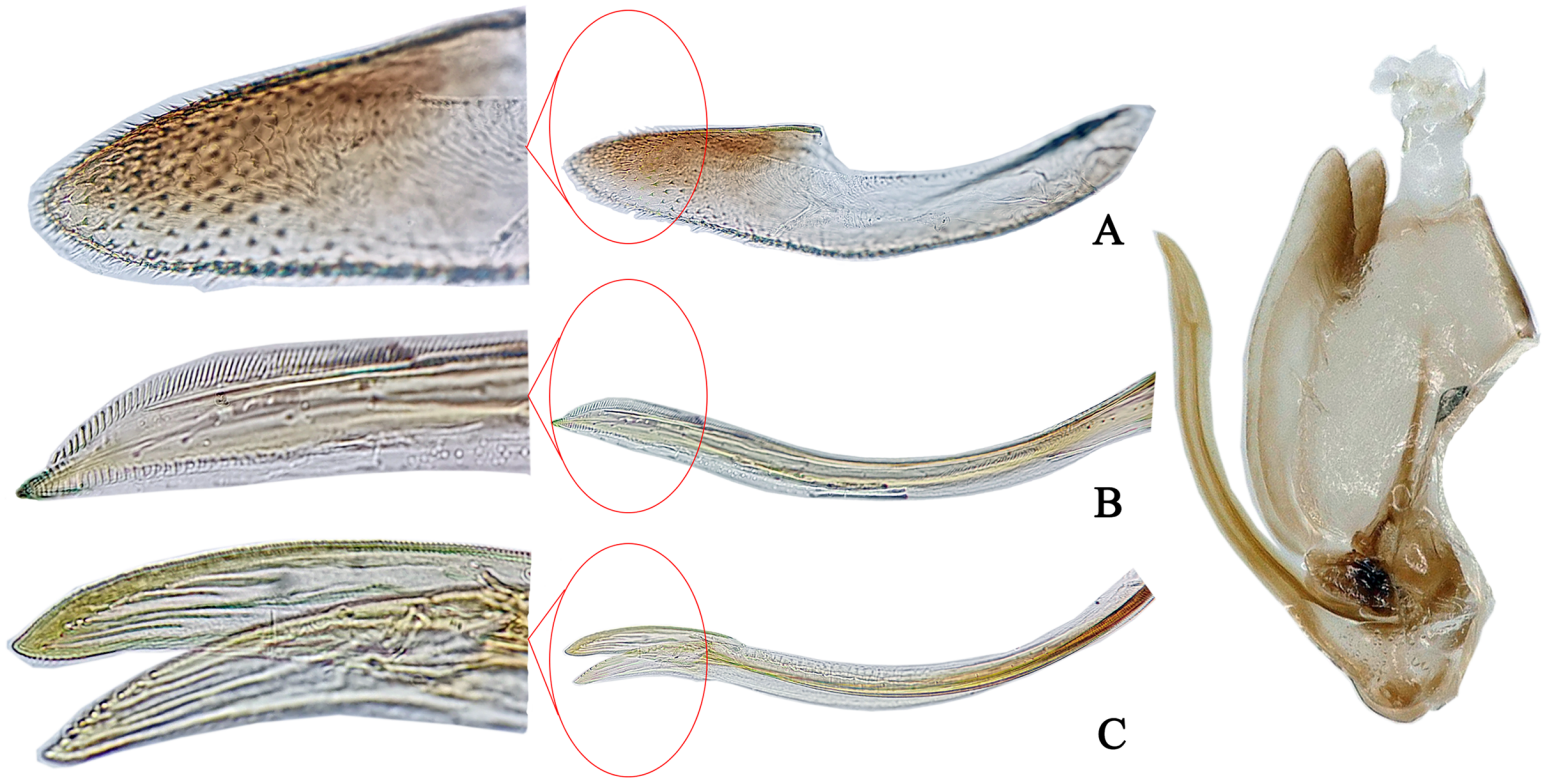
The morphological characteristics of female *E*. (*Eupteryx*) *minuscula*. (A) Valvula III. (B) Valvula I. (C) Valvulae II.

**Female genitalia.** First valvula of ovitositor tapered from base to apex with subapical ventral ‘heel’ and strigate dorsal sculpture (Fig. 2B); second valvulae asymmetrical with jaggies on dorsal margin apically (Fig. 2C); third valvula with several comb teeth on dorsal margin apically (Fig. 2A).

**Measurement.** Body length ♂2.7–3.1 mm; ♀3.1–3.3 mm.

**Specimen examined.** CHINA: 32♂♂, 69♀♀, Huajiang, 2019, coll. Zhouwei Yuan & Chao Tan.

**Distribution.** China (Sichuan, Shaanxi, Hubei, Jiangsu, Gansu, Guizhou), Far East of Russia, North Korea, Japan.

**Host plant.** *Artemisia* (*Asteraceae*).

### *Eupteryx* ( *Stacla*) *gracilirama* Hou, Zhang & Huang, 2016

*Eupteryx* (*Stacla*) *gracilirama* Hou, Zhang & Huang, 2016: 597

**Figure 3:**
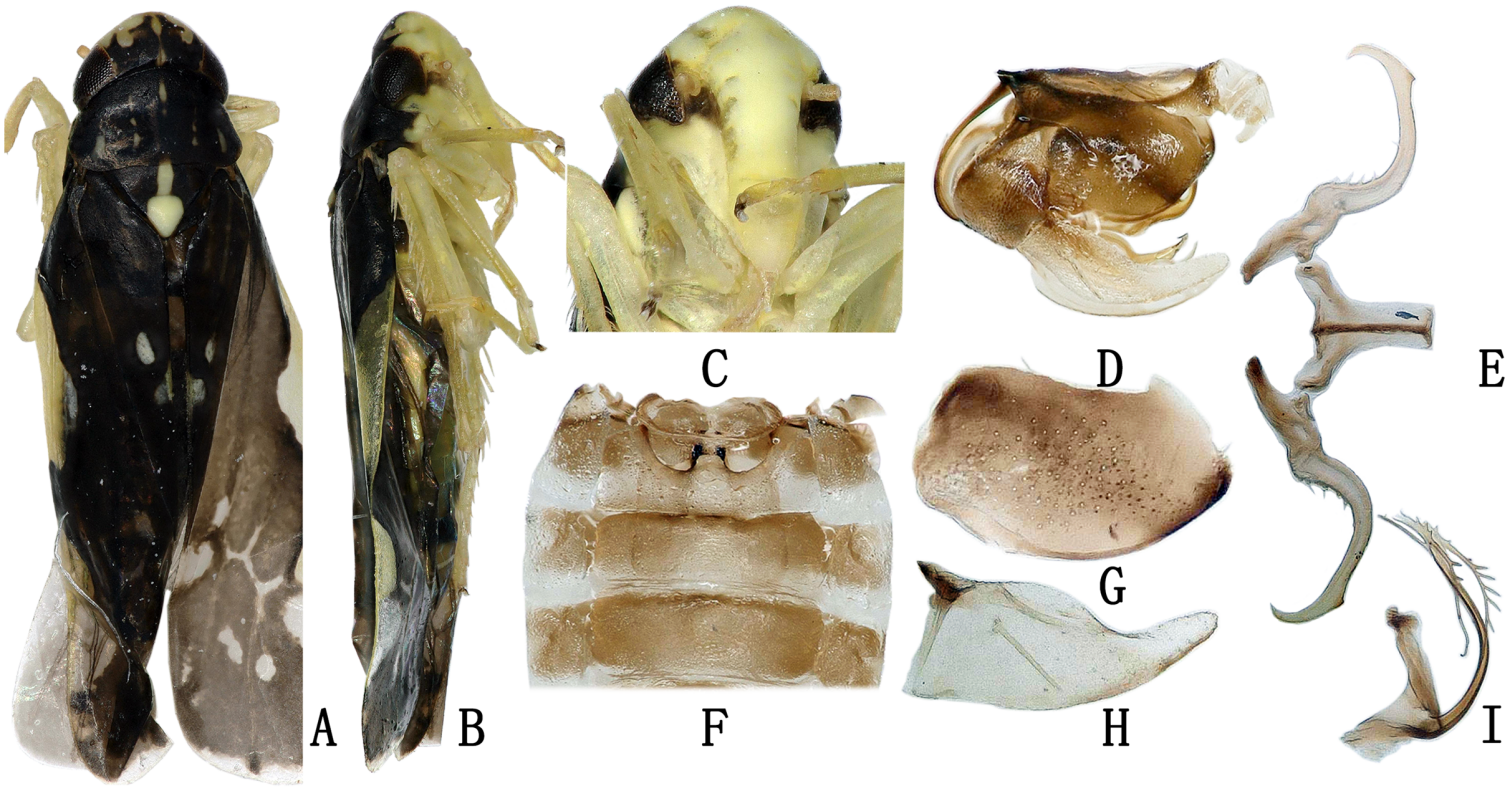
The morphological characteristics of male *E*. (*Stacla*) *gracilirama*. (A) Dorsal view. (B) lateral view. (C) Face. (D) Pygofer, lateral view. (E) Connective & styles. (F) Abdominal apodemes. (G) Pygofer lobe. (H) Subgenital plate. (I) Aedeagus, later view.

**Description.** Body dull yellow. Vertex and pronotum dark brown to black, with symmetrical yellow spots and stripes. Vertex with three yellow spots anteriorly and one posteriorly. Eyes black. Pronotum with five nearly parallel longitudinal yellow stripes. Mesonotum with dark brown lateral triangles and milky yellow apex (Fig. 3A). Forewing dark, with two yellowish patches in basal half and several whitish patches in apical half (Fig. 3B). Abdomen brown in dorsal view and light yellow in ventral view, except for darker apical segments (Fig. 3C). Abdominal apodemes extending to 5th sternite (Fig. 3F).

**Male genitalia.** Hind margin of pygofer lobe slightly produced, with several small rigid setae apically, and row of rigid setae at baso-lateral angle (Figs. 3D, 3G). Subgenital plate expanded basally, with row of microsetae at upper margin apically (Fig. 3H). Style with foot-like apex (Fig. 3E). Aedeagal shaft with pair of long branched apical processes extending based to lower half of shaft; smaller branches forming comb-like structure on ventral surface of each process (Fig. 3I). Connective Y-shaped, with lateral arms little longer than in previous specie; stem well developed (Fig. 3E).

**Figure 4:**
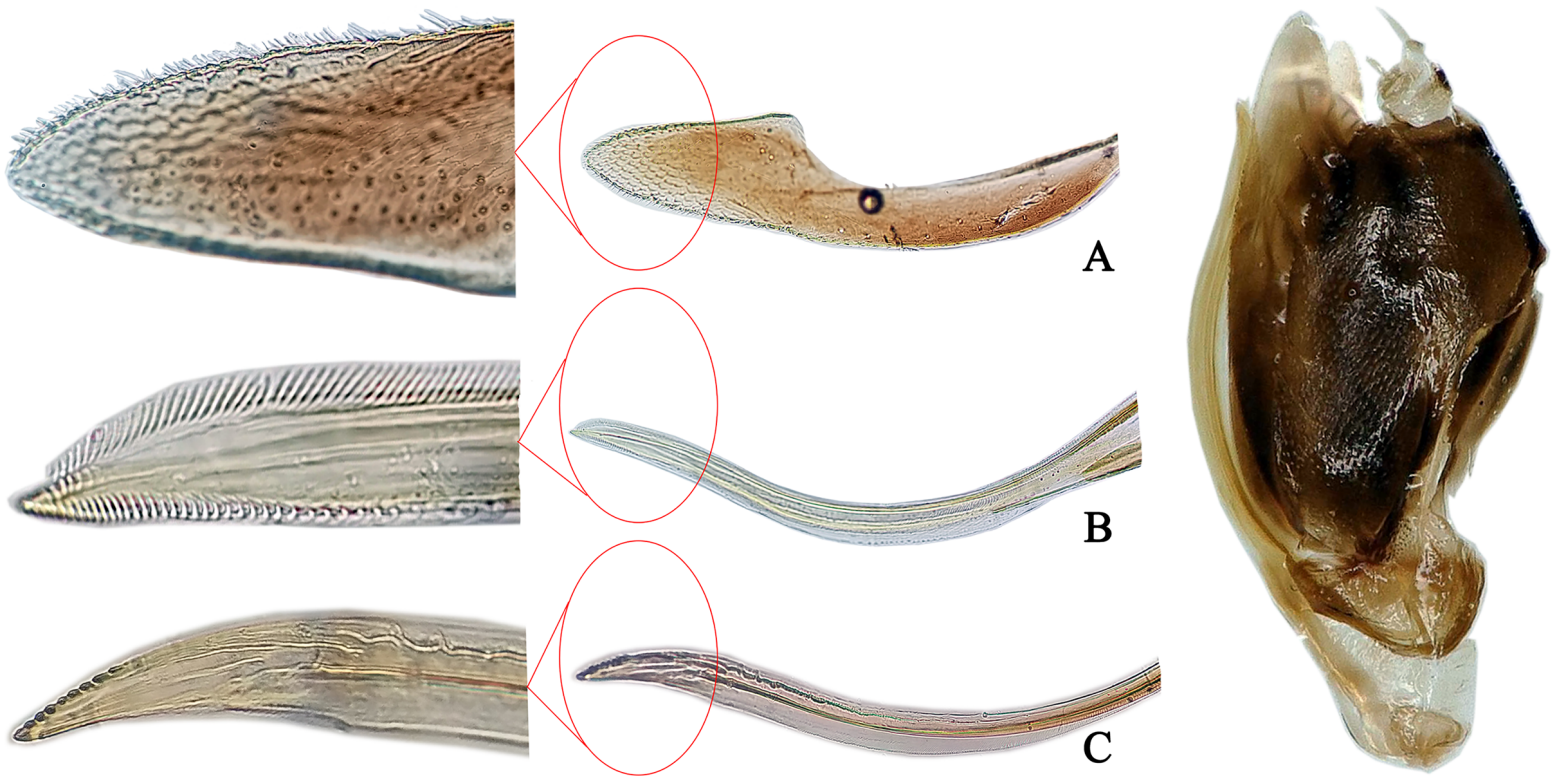
The morphological characteristics of female *E*. (*Stacla*) *gracilirama*. (A) Valvula III. (B) Valvula I. (C) Valvulae II.

**Female genitalia.** Female ovipositor similar to *E. minuscula* (Figs. 4A-C).

**Measurement.** Body length ♂3.06 mm; ♀3.07 mm.

**Specimen examined.** CHINA: 11♂♂, 9♀♀, Shibing, 2019, coll. Zhouwei Yuan, Xiaowei Yuan & Chao Tan.

**Distribution.** China (Yunnan, Guizhou).

**Host plant.** *Lamiaceae, Artemisia* (*Asteraceae*).

### Genome organization and composition

**Figure 5:**
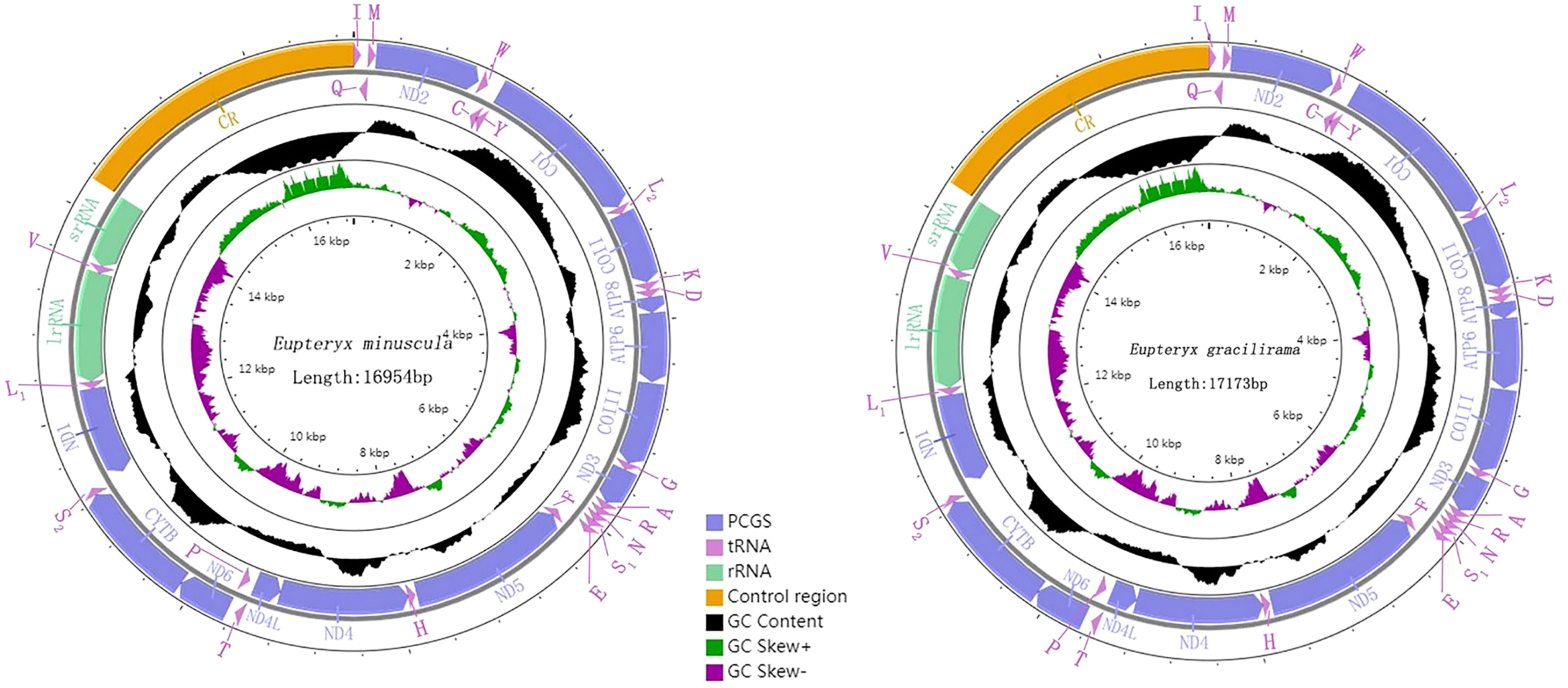
Organization of the complete mitogenome of *E*. (*Eupteryx*) *minuscula* and *E. (Stacla) gracilirama* protein-coding genes and codon usage.

The complete mitogenomes of *E. minuscula* (Genbank: MN910279) and *E. gracilirama* (Genbank: MT594485) are double-stranded plasmids with 16,944 bp and 17,173 bp respectively (Figs. 5). Both of them contain the usual 13 PCGs, 22 tRNA genes, two rRNA genes, and a control region. The genome organization and nucleotide composition of the two studied species are similar to *Limassolla lingchuanensis*, *Mitjaevia protuberanta*, *Empoascanara sipra* and *Parathailocyba orla* (Yuan et al., 2020; Chen, Li & Song, 2021; Jiang et al., 2020; Tan et al., 2020). In the *E. minuscula* mitogenome, gene overlaps were found at 11 gene junctions and involved a total of 41 bp, the longest 10 bp overlapping located between *cox3* and *trnG*; intergenic spacer sequences were found at 12 gene junctions and involved a total of 72 bp, the longest 20 bp intergenic spacer sequences were located between *nad5* and *trnH*. The mitogenome of *E. gracilirama* is relatively compact, with gene overlaps at 13 gene junctions, involving a total of 87 bp. The longest overlap of 41 bp occurs between *cox2* and *trnK*. Genetic interval at 13 gene junctions, involving a total of 116 bp. The longest interval of 48 bp occurs between *trnL2* (CUN*)* and *cox2* (Table 2).

**Table 2:** Annotations of the mitogenomes of *Eupteryx minuscula* (EM) and *Eupteryx gracilirama* (EG).

Gene	Location		Size		Intergenic		Start codon		Stop codon		Strand
					nucleotides										
	EM	EG	EM	EG	EM	EG	EM	EG	EM	EG	EM/EG
*trnI*	1–65	1–70	65	70							N
*trnQ*	63–131	68–136	69	69	−3	−3					J
*trnM*	131–199	136–204	69	69	−1	−1					N
*nad2*	200–1,159	205–1,182	960	978			ATA	ATA	TAA	TAG	N
*trnW*	1,179–1,244	1,187–1,253	66	67	19	4					N
*trnC*	1,237–1,297	1,246–1,306	61	61	−8	−8					J
*trnY*	1,300–1,364	1,313–1,378	65	66	2	6					J
*cox1*	1366–2,901	1,380–2,918	1,536	1,539	1	1	ATG	ATG	TAA	TAA	N
*trnL2*	2,905–2,970	2,914–2,980	66	67	3	−5					N
*cox2*	2,971–3,649	3,029–3,700	679	672	0	48	ATT	ATA	T	TAA	N
*trnK*	3,650–3,721	3,660–3,731	72	72	0	−41					N
*trnD*	3,722–3,786	3,734–3,795	65	62	0	2					N
*atp8*	3,786–3,938	3,796–3,948	153	153	−1	0	TTG	TTG	TAA	TAA	N
*atp6*	3,932–4,582	3,942–4,592	651	651	−6	−7	ATG	ATG	TAA	TAA	N
*cox3*	4,587–5,375	4,597–5,374	789	778	4	4	ATG	ATG	TAG	T	N
*trnG*	5,366–5,425	5,375–5,436	60	62	−10	0					N
*nad3*	5,428–5,781	5,437–5,790	354	354	2	0	ATT	ATT	TAA	TAA	N
*trnA*	5,782–5,843	5,796–5,858	62	63	0	5					N
*trnR*	5,847–5,914	5,857–5,924	68	68	3	−2					N
*trnN*	5,913–5,977	5,923–5,987	65	65	−2	−2					N
*trnS1*	5,977–6,041	5,987–6,053	65	67	−1	−1					N
*trnE*	6,048–6,114	6,075–6,139	67	65	6	21					N
*trnF*	6,115–6,178	6,141–6,204	64	64	0	1					J
*nad5*	6,179–7,829	6,205–7,855	1,651	1,651			TTG	ATT	T	T	J
*trnH*	7,850–7,911	7,874–7,937	62	64	20	18					J
*nad4*	7,911–9,230	7,937–9,256	1,320	1,320	−1	−1	ATG	ATG	TAA	TAA	J
*nad4L*	9,224–9,499	9,250–9,525	276	276	−6	−7	ATG	ATG	TAA	TAA	J
*trnT*	9,502–9,568	9,528–9,592	67	65	2	2					N
*trnP*	9,569–9,634	9,593–9,658	66	66							J
*nad6*	9,637–10,131	9,661–10,155	495	495	2	2	ATT	ATA	TAA	TAA	N
*cytb*	10,124–11,260	10,148–11,284	1,137	1,137	8	−8	ATG	ATG	TAG	TAA	N
*trnS2*	11,259–11,323	11,287–11,351	65	65	−2	2					N
*nad1*	11,324–12,256	11,351–12,283	933	933	0	−1	ATT	ATT	TAA	TAA	J
*trnL1*	12,257–12,322	12,284–12,354	66	71							J
*rrnL*	12,323–13,483	12,355–13,519	1,161	1,165							J
*trnV*	13,484–13,549	13,520–13,584	66	65							J
*rrnS*	13,550–14,283	13,585–14,321	734	737							J
AT-rich region	14,284–16,945	14,322–17,173	2,662	2,852							

The nucleotide composition of the whole mitogenome of *E. minuscula* and *E. gracilirama* was as follows: (A) 43.6% and 44.9%, (T) 35.2% and 35.6%, (G) 9.8% and 8.4%, (C) 11.4% and 11.1%, the AT content was 78.8% and 80.5 %, the GC content was 21.2% and 19.5% respectively, thus exhibiting a strong A/T bias. Besides, AT skew, and GC skew were calculated for the mitogenomes of *E. minuscula* and *E. gracilirama* (Table 3).

**Table 3:** Skewed nucleotide compositions of *Eupteryx minuscula* and *Eupteryx gracilirama* mitogenomes.

Region	A (%)		T (%)		G (%)		C (%)		AT (%)		GC (%)		AT skew		GC skew	
	EM	EG	EM	EG	EM	EG	EM	EG	EM	EG	EM	EG	EM	EG	EM	EG
**Whole**	43.6	44.8	35.2	35.6	9.8	8.4	11.4	11.1	78.8	80.4	21.2	19.5	0.107	0.114	−0.075	−0.138
**PCGs**	42.6	44.2	33.5	34.1	12.8	9.3	11.1	12.4	76.1	78.3	23.9	21.7	0.12	0.129	0.071	−0.143
**tRNA**	41.4	42.9	37.1	37.3	9.8	8.7	11.7	11.1	78.5	81.2	21.5	19.8	0.055	0.7	−0.088	−0.121
**rRNA**	47.1	47.8	35.4	35.9	6.8	6	10.7	10.3	82.5	83.7	17.5	16.3	0.142	0.142	−0.223	−0.264
**CR**	46.5	47.9	40.5	401	7.2	4.8	5.8	7.2	87	88	13	12	0.069	0.089	0.108	−0.2

Both of *E. minuscula* and *E. gracilirama* mitogenomes contained 13 PCGs, the total length is 10,934 bp and 10,937 bp, respectively (Table 2). Among the 13 PCGs, the largest is *nad5* gene and the smallest is *apt8* gene. The nine genes (*cox1*, *cox2*, *cox3*, *atp6*, *atp8*, *nad2*, *nad3*, *nad6*, and *cytb*) are located on the J-strand, whereas the other four genes (*nad1*, *nad4*, *nad4L* and *nad5*) are located on the N-strand. The proportion of A, T, G, C is 42.6%, 33.5%, 12.8%, 11.1% in *E. minuscula* mitogenome and 44.2%, 34.1%, 12.4%, 9.3% in *E. gracilirama* mitogenome, the A+T content of the PCGs in *E. minuscula* mitogenome (76.1%) is lower than in *E. gracilirama* mitogenome (78.3%). The start and stop codons of the 13 PCGs are shown in Table 2.

Most of PCGs started with ATN, except for some mitogenome. In *E. minuscula* mitogenome, all 13 PCGs utilize ATG (*cox1*, *cox3*, *atp6*, *nad4*, *nad4L*, *cytb*), ATT (*cox2*, *nad1*, *nad3*, *nad6*), TTG (*atp8*, *nad5*) and ATA (*nad2*) as start codon. Nine PCGs (*nad1*, *nad2*, *nad3*, *nad4*, *nad4L*, *nad6*, *cox1*, *atp6*, *atp8*) has TAA as stop codon, whereas the *cox2* and *nad5* genes use a single T, and the *cox3*, *cytb* genes use TAG. However, in *E. gracilirama* mitogenome, six PCGs are terminated by ATG (*cox1*, *cox3*, *atp6*, *nad4*, *nad4L* and *cytb*), three PCGs are terminated by ATT (*nad3*, *nad5*, *nad1*), *nad2*, *nad6*, *cox2* used ATA, *atp8* utilize TTG. Ten genes use TAA as a stop codon, the *nad2* gene utilize TAG, and the *cox3* and *nad5* genes use a single T.

**Figure 6:**
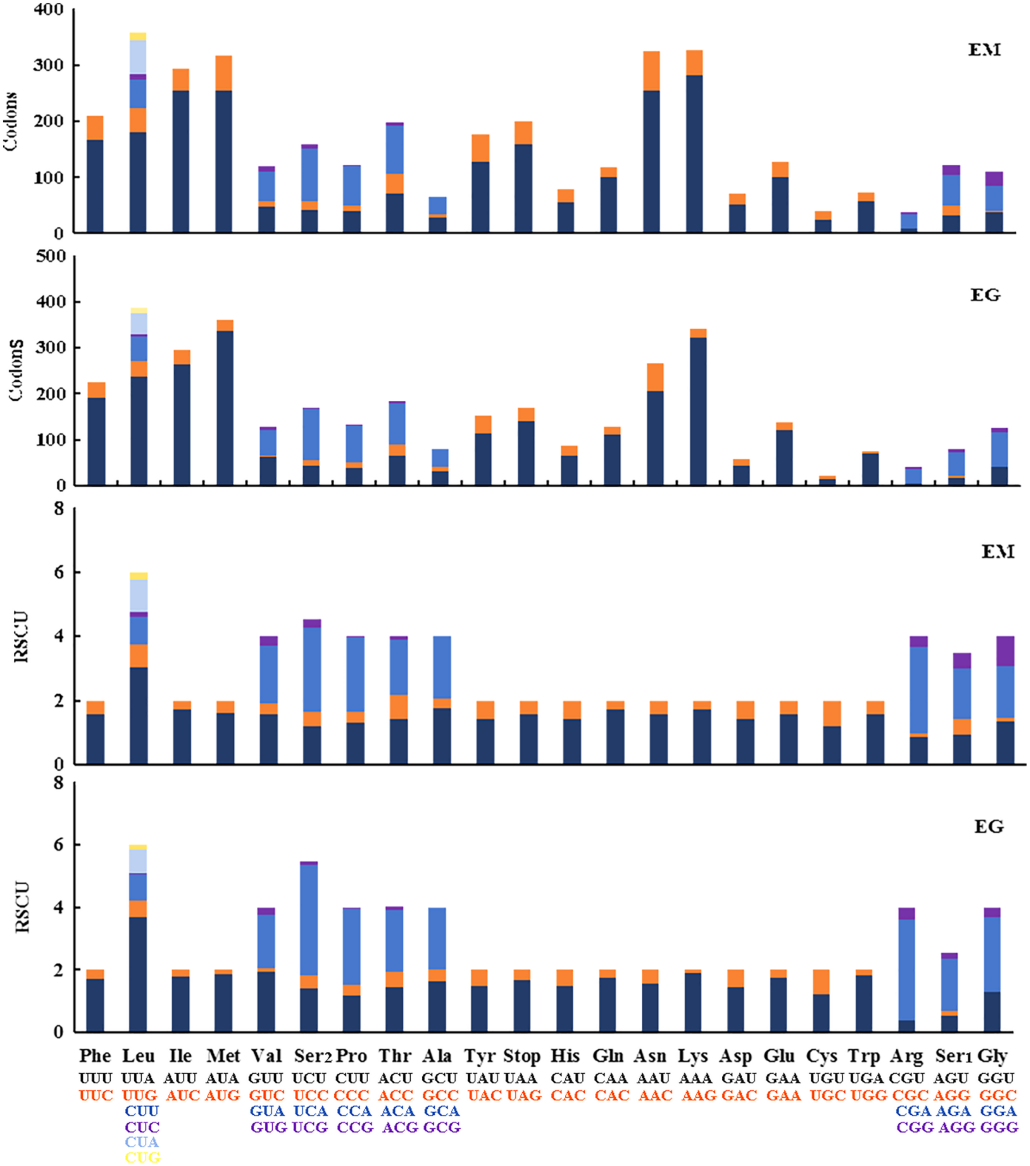
Relative synonymous codon usage (RSCU) and codon distributions (Codons) in the mitogenomes of *E*. (*Eupteryx*) *minuscula* (EM) and *E*. (*Stacla*) *gracilirama* (EG).

The 13 PCGs in *E. minuscula* comprised 3,644 codons and those in *E. gracilirama* mitogenome comprised 3,645 codons. The behavior of the codon families were determined in the PCGs, which showed the codon usage in the *E. minuscula* mitogenome, which the five most frequently used codons are Leu, Lys, Asn, Met, and Ile. *E. gracilirama* mitogenome, where the five most frequently used codons are Leu, Met, Lys, Ile, and Asn (Fig. 6). Relative synonymous codon usage (RSCU) is summarized in Fig. 6, indicating that the most frequently utilized amino acids are Leu, Lys, Ile, Asn and Met. One newly sequenced includes 63 available codons but the codons Ala (GCG) are absent in *E. minuscula*, another includes 61 available codons but the codons Ala (GCG), Arg (CGC) and Gly (GGC) are absent in *E. gracilirama*.

### Transfer RNA and ribosomal RNA genes

**Figure 7:**
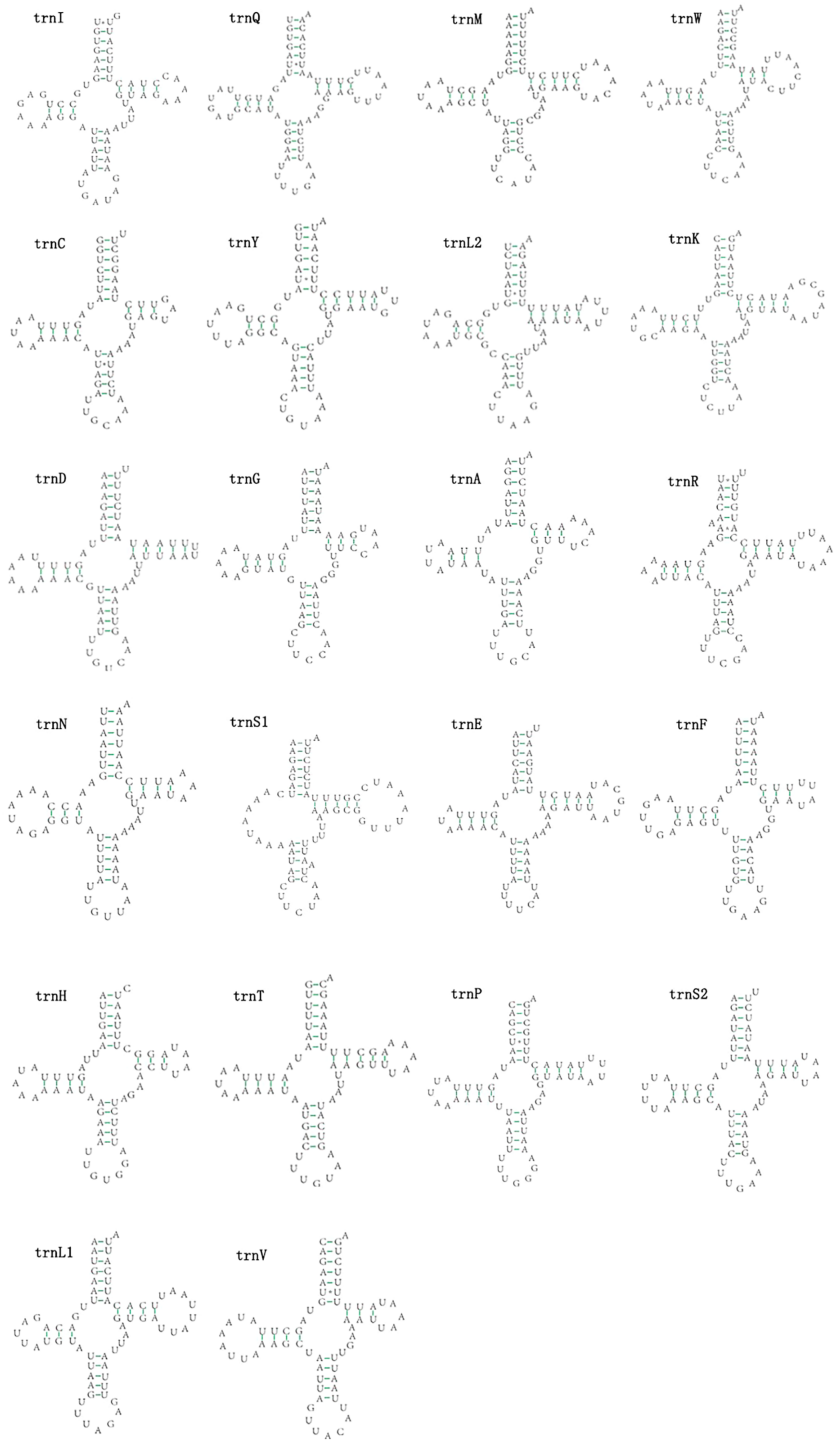
Predicted secondary cloverleaf structure for the tRNAs of *E*. (*Eupteryx*) *minuscula*.

**Figure 8:**
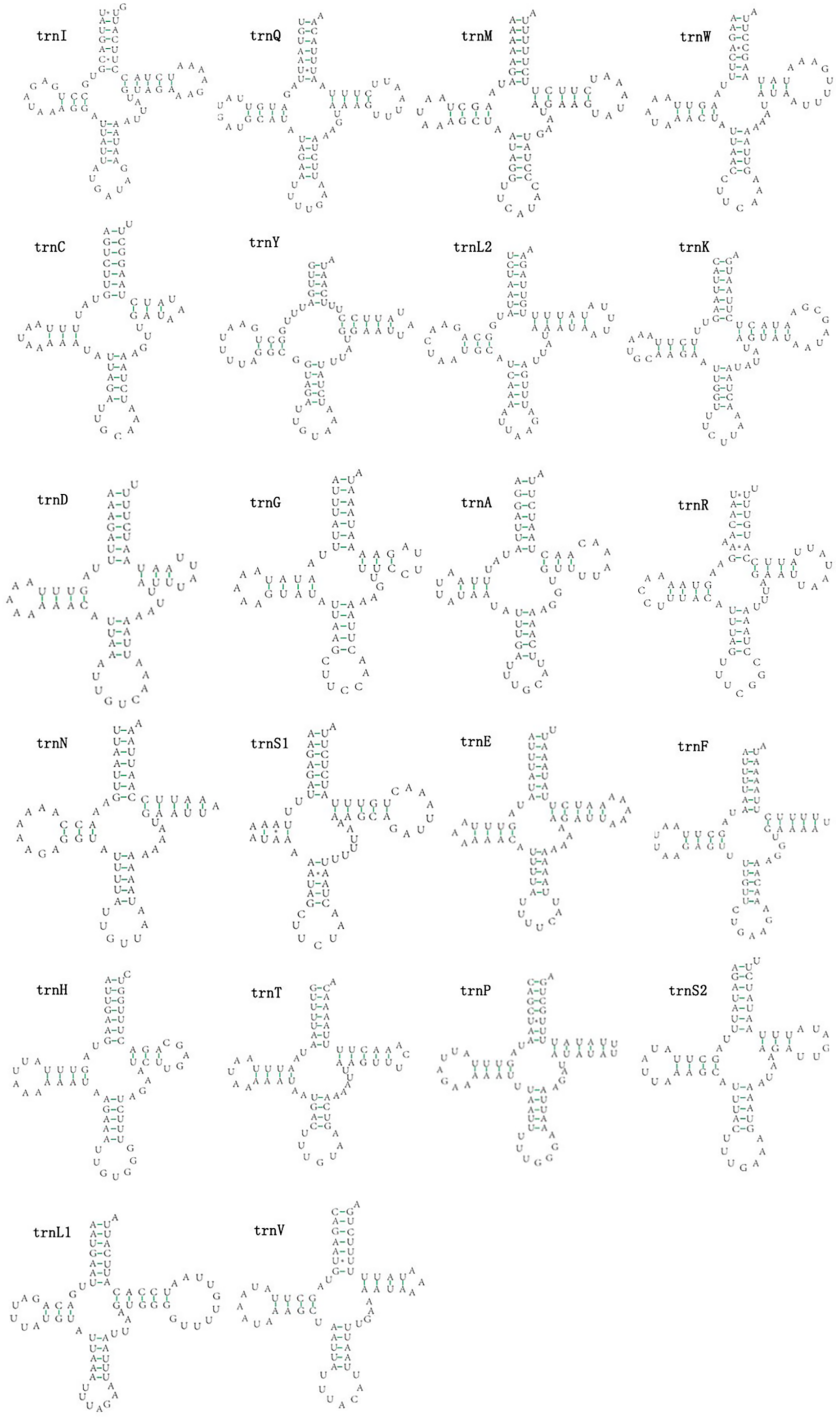
Predicted secondary cloverleaf structure for the tRNAs of *E*. (*Stacla*) *gracilirama*.

The mitogenomes of *E. minuscula* and *E. gracilirama* contain 22 tRNAs. Fourteen tRNA genes are oriented on N-strand, others are transcribed on the J-strand (Table 2). The tRNA of the two species have a positive AT and negative GC skew (Table 3), and range from 60 to 72 bp. In *E. minuscula* mitogenome, the lengths ranged between 60 bp (*trnG*) and 72 bp (*trnK*). In *E. gracilirama* mitogenome, the largest is 72 bp (*trnK*) and the smallest is 61 bp (*trnC*). In *E. minuscula*, *trnS1* (AGN) lacks the dihydrouridine arm forming a simple loop, other tRNAs are not, which lacks as commonly found in other insect mitogenomes (Cameron, 2014; Li et al., 2017). The total number of unmatched base pairs found was 40 and 31 in *E. minuscula* and *E. gracilirama*, and types of mismatches were A-A, A-C, U-U, C-U and G-U (Figs. 7, 8). Moreover, these two mitogenomes have *trnR* with an unpaired base in the acceptor stem. In *E. minuscula*, *16sRNA* gene (1,161 bp) is located between *trnL1* (UUR) and *trnV*, *12sRNA* gene (734 bp) between *trnV* and A+T rich region. In *E. gracilirama*, *16sRNA* gene (1,165 bp) is located between *trnL1* (UUR) and *trnV*, *12sRNA* gene (737 bp) between *trnV* and A+T rich region. The rRNA genes had a positive AT skew and negative GC skew, and presented a heavy AT nucleotide bias, with A+T content 82.5% in *E. minuscula* and 83.7% in *E. gracilirama* (Table 3).

### A+T rich region

The A+T rich region is located between *12sRNA* and *trnI*, which is the longest in mitogenomes of *E. minuscula* and *E. gracilirama*. The A+T rich region of *E. minuscula* is from 14,284 bp to 16,945 bp, and 2,662 bp in length with an A+T content of 87.0%. The A+T-rich region of *E. gracilirama* is from 14,322 bp to 17,173 bp, and it measured 2,852 bp in length with an A+T content of 88.0%. The nucleotide composition of the A+T rich region of *E. minuscula* and *E. gracilirama* are as follows: (A) 47.9% and 46.5%, (T) 40.1% and 40.5%, (G) 4.8% and 7.2%, (C) 7.2% and 5.8%, respectively (Table 3).

### Phylogenetic relationships

**Figure 9:**
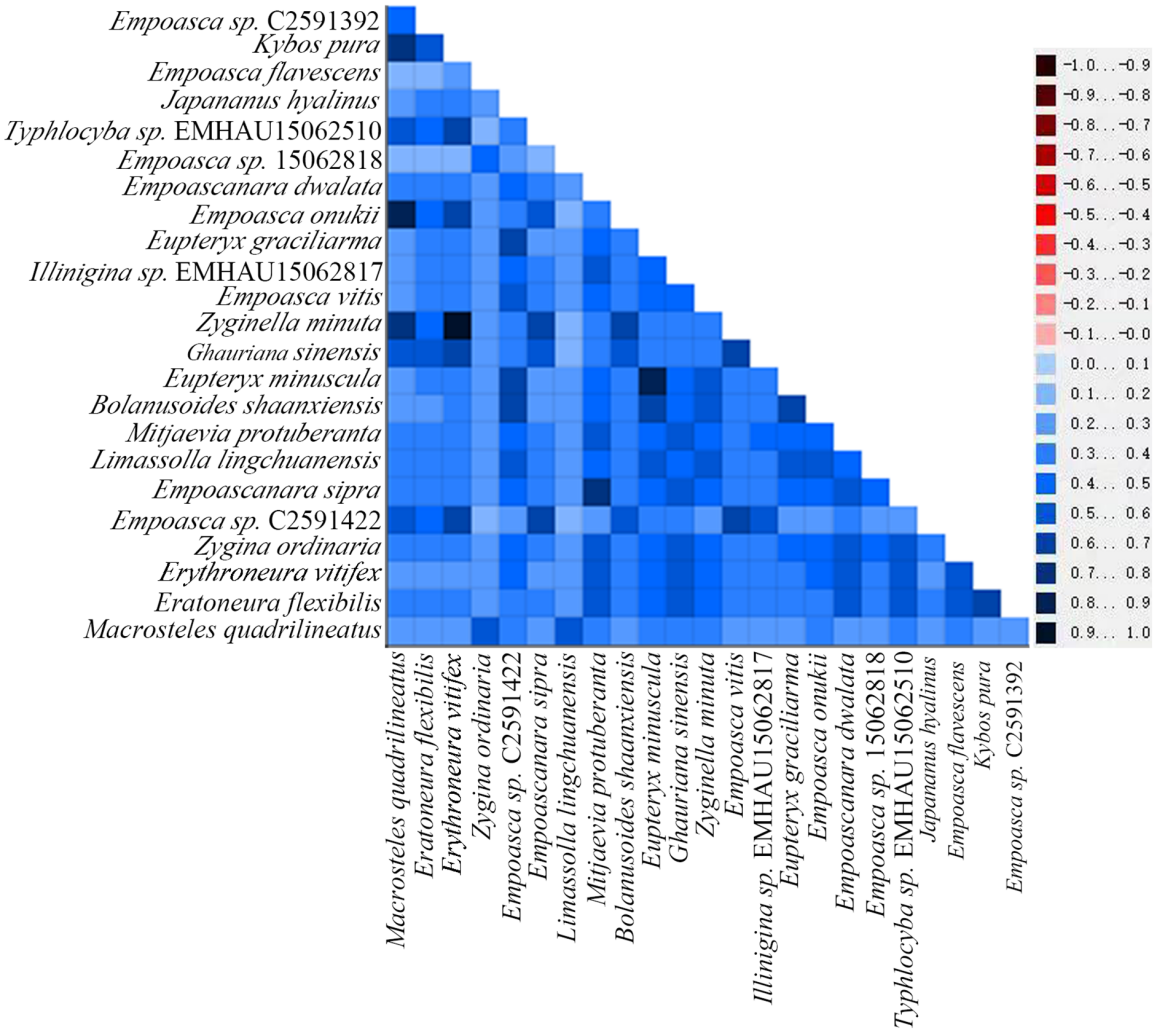
Heterogeneity of 13 protein-coding genes in the mitogenome of Cicadellidae. Differences in heterogeneity between sequences are represented by color, with dark red (−1) to dark blue (+1) representing differences from heavy to light.

By examining the base heterogeneity of mitogenome dataset used to construct phylogenetic tree, we can determine whether the underlying heterogeneity of each dataset will lead to significant errors during tree construction (Sheffield et al., 2009; Li et al., 2015; Timmermans et al., 2015). On the basis of the calculation results obtained from the AliGROOVE (Kück et al., 2014) software, the heterogeneity of PCG data sets in the mitogenome data of Cicadellidae is weak (Fig. 9). So, the dataset could be used to construct a phylogenetic tree.

**Figure 10:**
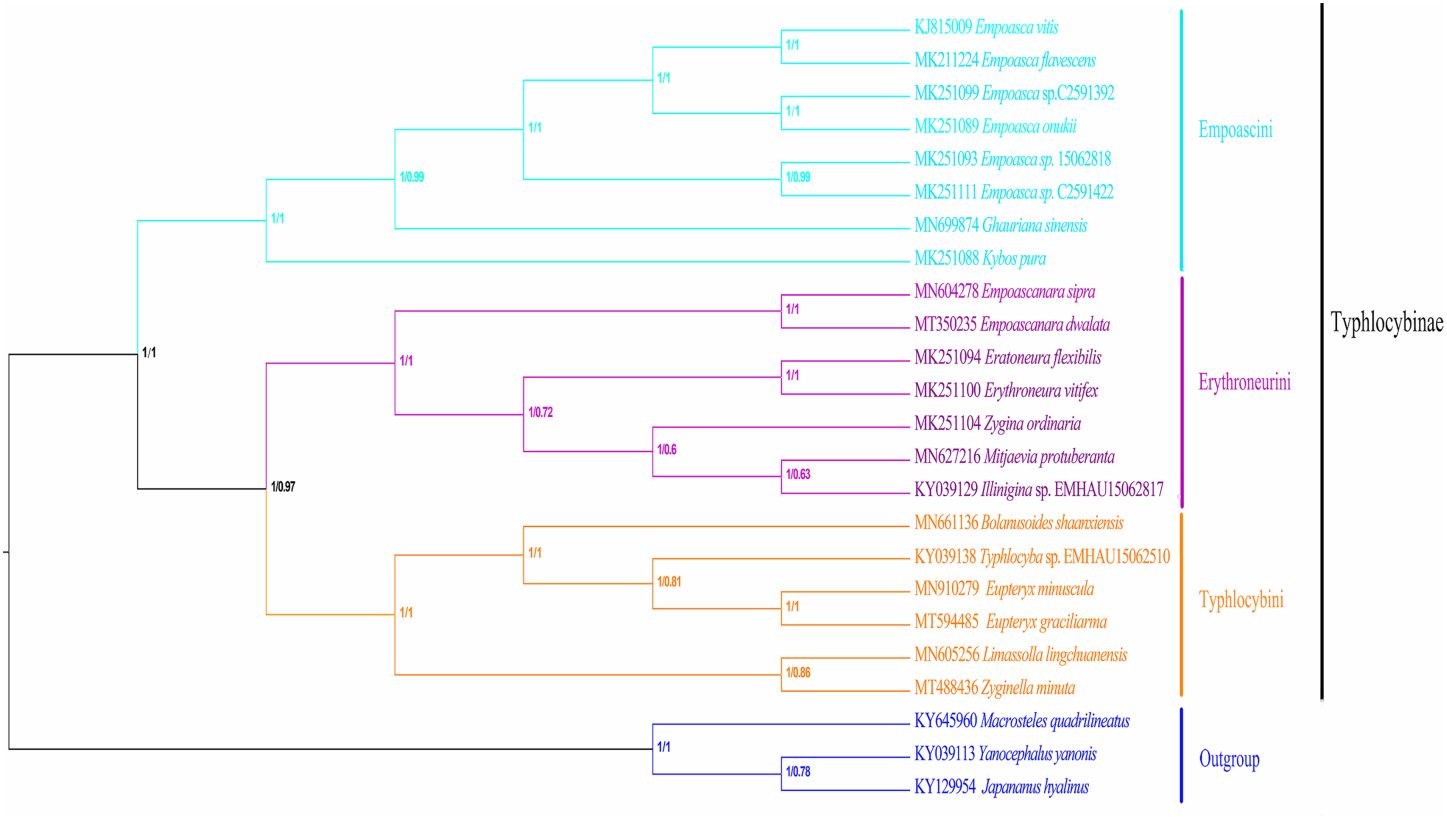
Phylogenetic tree inferred by maximum likelihood and maximum parsimony based on the protein-coding genes.

In recent years, molecular sequencing technology has been widely used in phylogenetic analysis, which can test and verify the results of different levels of more morphology based traditional classifications. Within Typhlocybinae, only a few studies have used the combination of morphological characteristics and molecular data to construct phylogenetic relationships. Song, Yuan & Li (2020) used 13PCGs of eight species of Typhlocybinae to construct a phylogenetic tree and obtained the following topology: Empoascini + (Typhlocybini + (Erythroneurini + Zyginellini)). In this study, the phylogenetic tree (Fig. 10) was erected based on 13 PCGs mitogenomes data of 21 leafhopper species of Typhlocybinae. The most phylogenetic relationships in this subfamily were highly supported. The both tribes of Typhlocybini and Zyginellini are sister groups, and merged into a large branch. The result supports the combination of Typhlocybini and Zyginellini as a tribe. And the results obtained the following topology: Empoascini + ((Typhlocybini + Zyginellini) + Erythroneurini). It differs slightly from the traditional classification system (Fig. 10). Because the limited number of species data are available in our study, so adding more Typhlocybine species and representatives of other major leafhopper groups in Cicadellidae will be needed to solve the problem of the phylogenetic relationship of Typhlocybinae very well.

## Discussion

In this study, we redescribed the external features of the two species. The main diagnostic features of the genus *Eupteryx* are the following: male hind margin of pygofer lobe with or without sclerotized process; several enlarged macrosetae forming group at baso-ventral angle of lobe; subgenital plate extremely broad at base, with single long macroseta on outer margin subbasally; aedeagus usually with pair of processes at apex. *E. minuscula* and *E. gracilirama* are representatives of two subgenera, *Eupteryx* and *Stacla* respectively. The main difference between the two species is the shape of pygofer lobe, which has well developed ventral process in *E. minuscula* or without process in *E. gracilirama*. Style, subgenital plate, Y-shaped connective, and aedeagus (with paired long apical processes) are comparable in both species and differentiated only in minor features.

In addition, we analyzed the mitogenomes composition and structure of *E. minuscula* and *E*. gracilirama. And the location, secondary structure, PCGs, tRNA genes, rRNA genes, and control regions, and compares them with other Typhlocybine mitogenomes. These two species closely favor those of most other sequenced leafhoppers in various structural and compositional aspects. Moreover, Phylogenetic trees inferred by Maximum likelihood and Maximum parsimony based on the PCGs of 21 species. Empoascini and Erythroneurini were recovered as monophyletic while Zyginellini and Typhlocybini gathered into a single branch. The conclusion of leafhopper phylogeny of this paper supports Dietrich’s view (2013), Zyginellini is a junior synonym of Typhlocybini, that is to say, the two tribes should be placed into the same taxon as a monophyletic group. There is some different from the traditional classification system, the tribe Zyginellini evolved from the tribe Typhlocybini and was in a higher evolutionary position in the subfamily Typhlocybinae (Zhang, 1990).

## Conclusions

Based on the current and previous studies, the classification of the tribes of Typhlocybinae is not yet fully resolved with respect to Typhlocybini and Zyginellini, because of the very limited molecular data of the leafhopper species of Zyginellini in this study. The new data provided here will facilitate future comparative studies of Typhlocybine leafhopper mitogenomes. So, more sequencing data is needed to build a more complete phylogenetic tree or combine the traditional morphological classification for the better phylogenetic analysis of Typhlocybinae.

## Supplemental Information

10.7717/peerj.12501/supp-1 Supplemental Information 1 Taxonomic information and GenBank accession numbers for the species used in this study.

10.7717/peerj.12501/supp-2 Supplemental Information 2 Annotations of the mitogenomes of *Eupteryx minuscula* (EM) and *Eupteryx* *gracilirama* (EG).

10.7717/peerj.12501/supp-3 Supplemental Information 3 Skewed nucleotide compositions of *Eupteryx minuscula* (EM)and *Eupteryx gracilirama*(EG) mitogenomes.

10.7717/peerj.12501/supp-4 Supplemental Information 4 Annotations of the whole mitochondrial genomes of Eupteryx (Eupteryx) minusula.

10.7717/peerj.12501/supp-5 Supplemental Information 5 Annotations of the whole mitochondrial genomes of Eupteryx (Stacla) gracilirama.

## Additional Information and Declarations

### DNA Deposition

The following information was supplied regarding the deposition of DNA sequences: The group II intron/IEP sequences are available at GenBank: MN910279 and MT594485.

### Data Availability

The following information was supplied regarding data availability: The raw measurements are available in the Supplementary Files.
