# Supplementary material for: Characterization of the morphology and complete mitochondrial genomes of Eupteryx minusula and Eupteryx gracilirama (Hemiptera: Cicadellidae: Typhlocybinae) from Karst area, Southwest China
Source: PeerJ. 2021 Nov 24;9:e12501. doi: 10.7717/peerj.12501 (PMC8627127; doi:10.7717/peerj.12501)
Supplement: Supplemental Information 4 [file peerj-09-12501-s004.docx]

TGTGAAGTGCCTGAGAAAAGGATTATTATGATAGAATAAATTATGTAGAAAACCTACTTTCATTGTGAATTAAAGAATTAAACTTTCCTTTAGAATTCAAAATTCCATATGCATCATAACATCTAATCACAAAAAGGTAAGCTAATAAAGCTATTAGGTTCATACCCTGCGAATAGAAGTACAAATCTTCTTCTTTTTAATAAATTTTAATTCAACAAAAATATTGTTTTACACCACCATATTGATAGGGGTCACTATATCAATTAGATCAAATAATTGAATCATAATATGATGTGGATTAGAAATTTCATTAGTATCATTTATTCCATTAATAGTTTCTAAATTAATTATATCGTCAGAGTCCTCTATTAAATACTTTATTGTTCAAAGAATTAGATCATCTATATTAATGCTGGGAATTACATTCATAATTATAAAGGGAGATTATAATTATGAATACGCAATAATGACCGCTTTATTGATCAAAACAGGGGTTTGCCCTTTCCATAATTGAGTACTAACAGTACTTGAGGGATTAGATTTCTATATAATCTTGATTATGCTGACAATTAATAAAATTGCACCATTAACTATAATGAGATACATTATAAGCTCAATAGTTTTAATTATTTACTTAACCACTATGATGGGAGCAATCATAGGTCTAAATCAAAATTCATTAAAGAAATTAATAGGTTATTCATCGATTTTTAATATAGGGTTAATTATAGCAGCCTTAAAAATAAATTGAATGTGAATATTATACCTACTAATCTATTCATTCCTATTATTTATACTATCAATATTTATAAATATAAACAATTTAAACCTTATTAATCAAATTATATTCTCAAGATCTATATTAATATCTATATGTTTATGAATTAACGTATTATCTATAGGTGGTATACCCCCTTTAATAGGATTCTCAATTAAATATTTAGTAGTAATATTTTTGGTGAGAACTAAAATAAATATGATAGTTTTTGTTATAATGATTTCATCATTATTAGTAATATTCTTTTACTTACGACTAACATTTATTAGATCAATAAATAATTTTATTACAAATAAAATAAAATTATTCAATTTGAAAGAAATTTCGCTATGAATAATTGTGGTTAACACATTAACACTACCAATGATATTAATAGTATAAATTTACTTATAGTCCTTTTAAGACTTTAAGTTAAATAAACTATTAACCTTCAAAGTTGAAAATATACTTCAATTTATAAGCCTTAGAACTACTCATTTTAAGATTTGCAATCTAATGTTTTTATTAAACTATAAGACCAATATTGAAAGGAATAACATTCCATAAGTAAATTTACAGTTTACTGCCTAAATTCAGCCATATCAACAATGAAAAAATGATTATTTTCTACTAATCATAAAGACATTGGAACGATATATTTTGTCTTCGGGGTATGAGCTGGAATATTAGGAATAATATTAAGAATATTTATTCGAATTGAATTAGCTCAACCAGGAGCTTTTTTAAATAATGACCAAACATACAATGTAATTGTTACTTCACATGCATTTATTATAATTTTCTTTATAGTTATACCAATCATAATTGGTGGTTTTGGAAATTGACTTCTTCCATTAATAATTGGTGCACCAGACATAGCTTTCCCACGAATAAACAATATAAGATTTTGATTACTACCACCATCACTAACATTGCTTTTAATTAGATCAGTAGTTGAAATAGGGGCAGGGACTGGATGAACAGTTTATCCTCCTTTAGCAAGTAATATTGCACACTCAGGAGCTAGAGTTGATTTAACTATTTTTTCATTACACTTAGCAGGTATTTCATCAATTTTAGGAGCAGTAAATTTTATTACAACAGTAATTAATATACGTACTAGATTAATAACATTAGATTCAACTCCATTATTTGTTTGATCAGTATTTATTACAGCTATTCTGTTATTATTATCATTACCTGTTCTAGCAGGTGCAATTACAATACTATTAACTGACCGAAATATTAATACTTCTTTCTTTGACCCATCAGGAGGAGGGGACCCGATTTTATATCAACATTTATTTTGATTCTTTGGTCACCCAGAAGTTTATATTTTAATTTTACCAGGATTTGGATTGATTTCACATATTGTAACCCAAGAAAGAGGTAAAACACAATCCTTTGGATATATTGGGATAGTGTATGCTATATTATCAATTGGTTTATTAGGGTTTGTAGTTTGAGCCCACCATATATTTACTGTTGGTATAGACGTTGACACACGTGCATATTTTACTTCAGCAACTATAATTATTGCAGTACCTACAGGTATTAAGGTATTTAGATGAATGGCTACAATAAATGGAATTGTAATCAAAAAAAGAATATCATTAATCTGATCATATGGGTTTGTGTTTTTATTCACAATAGGAGGATTAACAGGAATTATTTTATCTAATTCATCTATTGATATAGTATTACATGATACATATTATGTAGTAGCTCATTTTCATTACGTTTTATCAATAGGAGCTGTATTCGCTATTATAGCAGGTTTTATTCAATGATACCCTTTATTTACAGGTTTAACACTAAATCCTAAATTATTAAAAATTCAATTTGTAATAATATTTATTGGAGTTAATATAACATTTTTTCCTCAACATTTCTTAGGATTAAGAGGATTCCCACGACGATACTCTGATTACCCAGACGTATACTTGTCATGAAATATAGTTTCATCTATAGGAAGAAATATATCACTAATTAGAGTTTTAATAATAATATATGTTATTTGAGAAAGAATAGTAGTCAAACGGACTGTAATATTTAGAATGAATAATATTTCATCAATTGAATGACTTCAAGAAATGCCACCTAAGGAACATTCTTATAATGAATTACCATTTATCATAAAGTAATTTTCTAATGTGGCAGATAAATGCGCCAAACTTAAGATTTGTTAATAAATAATTTATATTTTTTTAGAAATTTCAAATTGATTTGGGTTAAATTTACAAAATGCTAATTCATCAATTATAGAGCAACTAATTATATTTCATGACCACACAATAATAATTGTGTTAATTATTACTATTATAGTAGGGTATATAATAATATTAATTATGATTAAAAGATTTAATAGACGATTTACTATAGAAAATCAAATAATTGAATTTACATGAACTGTATTACCAGCAGTGATACTTGTATTTATTGCATTACCATCTCTTAAAATTTTGTATATAATTGAAGAAACAGGTAAACCTTTAGTTTCAATTAAAACAATTGGTAATCAATGATTTTGATCCTATGAATATTCAGATTTTTCAAATATTATGTTTGATTCTTACATATCACCAATTAAGAATTCAACAATAAACCAAATTCGATTACTAGAAACAGATAATAAATTAGTAATTCCATATAATACTCAAACACGAATTTTAATTACATCTTATGATGTAATTCATTCTTGAACTATCCCAGTTTTAGGAATTAAAATTGATGCAGCTCCAGGACGAATTAATCAAGGAAATTTAATTATAACACGACCAGGATTATTCTTTGGTCAATGCTCAGAAATTTGTGGTGCAAACCATAGATTTATACCAATCATACTAGAAAGAGTTGGAATTAAATCATTTATTAACTGAATTAAAAATTATTCATTAAGTTTCTTAAATGCAAGAATTGGTCTCTTAAACTAAAATATAGTATAATAGCGAATACTCTTAATGAAAAGATTTAGTTTTAAAAAAAACGTTAATTTGTCAAGTTAAAATTATTAATTTTAATAATCTTTTTGCCACAAATATCTCCAATATGATGAATATCATTAATAATAATATTTATTATTACTATAATGATTTGTATTAACATAAATTACTTCAATTACAACAAAATATTAAAAATAAAAAAAATTGGTTACAAAAAAATAATAAACTGAATATGATAAATAATTTATTTTCCGTATTTGATCCATGTACAGGAATACTATCAATAAATTGATTAAGAACAATTATTTTTATAATAATAATGCCTTATAGAATATGAATTACAAATAATAAAATTTCATTCTTATTTAAAACAATTTTAAATAGACTTCATACAGAAATAAGTATATTAATAAAATTTAAAGGAACAACTCTAATCACAATCACATTGTTTACATTAATTTTTTATAATAATATTATAGGATTATTACCCTACATTTTTACTTCTTCAGCACACTTAGTTTTCTCATTAACAATAGCATTACCTATATGATTAGGGTTTATACTATATGGATGGATTAATAAAACTAATTATATGTTAACACATTTAGTACCAACTGGTACACCAGGAGTGTTAATACCATTTATAGTTTTAATTGAAACAATTAGAAACATTATTCGACCTGGGTCATTAGCTGTTCGATTAACAGCCAATATAATTGCTGGGCATCTATTAATATCATTATTAGGAAGAAACATCTCAATAATTATATTTATAATAACATTAACTGTTTTTATTATACTTATAATGTTTGAACTAGCAGTTGCACTAATTCAATCTTATGTATTTATAACACTAACTACATTGTATTCTAGAGAAATTTAAATTTATGAATAATCATCCATTTCATTTAGTAAATAAAAGACCTTGACCAATTACGGGGACTATTGGAATAATGTCAATATTATCCGGAATAGTTATATGATTTAGTAAACAACAAAATATCTTTATAATAGTAGGAATATTAATTGTTATTTTAACAATAATTCAATGATGACGAGACGTAACACGAGAAGCTAGATTCCAGGGACTTCACACTAGTAAAGTAATTTCATCTATAAAAATAGGTATAATTATATTTATTTTATCTGAAGTATTATTTTTCATAAGATTTTTCTGAGCATTTTTTCATAGAAGATTAGCACCACAAATAGAACTTGGTATACAATGACCACCTATAGGCATTGCAAGTTTTAACCCCATTAATATCCCCATGCTTAATACAATAATTCTGCTAAGATCAGGGATTACAATTACATGAGCTCATAATGCTTTAATTATAAAAAACTATACAAAAATAAATCAAGCAATAGTTATAACAATTGCATTAGGGATTTATTTTTCTATTCTTCAATTATATGAATATATTGAAGCACCATTTTGTATATCAGATTCAGTATACGGGTCAACATTTTTTTTAGCAACAGGTTTTCATGGTTTACATGTAATTATTGGTACAATTTTTATTGCTTGTATCTGCACAACGACACTTAAAACTTCACTTTTCAAAGGAACACCATGTAGGATTTGAAGCTTCAGCATGATACTGACACTTTGTAGATGTAGTCTGATTATTTTTATACACAAGAATTTATTGATGAGGAGGATATTTATTTAGTATAAAAAGTATGTTAAGCTTCCAACTTAAGGGTTTCCAATGAAAATAAATAATTATATTATCAATAAAACATTTAGTTGTAATTATAGTTATTATAATAGTTCTAATAGCCTTAATAATATTTGTTAGAAAAAAAACAATTCAAGATTGACAAAAATCTAACCCATTTGAATGTGGATTTAATTCAATAAGAATAAAACGATTACCATTCTCTATTCACTTCTTCTTGATTGCAGTTATTTTTCTTATTTTTGACATTGAAATCATTATTATTTTACCAATAACATTAACTTTAAAAAGATCATTTATAAGAATTTGAGTAATAACGTCAACATTATTTATTTTAATTTTATTAATAGGATTATACCATGAATGATTAAATGGAATACTAAAATGAACAACATAAAGGATTATATTTAATTATAATATTTGATTTGCATTCAAAAGGTGTTTCAAAAACTAATCTTAGAATAACAAAGAAGTAAAAATTACATTTAGTTTCGACCTAAAAATAGAATATAAAATTTATTCCATGTTTTAATTGAAACCAAAATAGAGGTATTTTATTGTTAATAAAAAAATTGAATAAAATTCCAATTAAAAGAGATCAAAATAAAAATAGCTTCTAACTAATTTTTAAAGCGGTTTAAATCCGTTTATCTCTTAATTATAATTCATATAGTTTATAAAACATTTTATTTTCATTAAAAAAAAAGATTAATGCATAATCTTATGAATTTATTTTAAGAAAATATTCACCTTTGTAACTTCAACACAAAACTCTCAACTTAAGCTATTAAAATAAATAAAAATAATAAATCCAATTATAAAAGATAATAAAAATATTTTTATATTTCCAAATATATAAAACTGTAATAGATTAGAAATTTTTAATAAATAAAAAGAAAAACCAGAAGAACCATAAAATTCCCCTCAATTTAAAGAATAGTTATACTTATAACTAAATAAATAAAAATTTATAAATAAGTAATAAGAATATCTGTATATTAATCATATATTAGAAAATAAATAATAAGAGTAATTAAATAGGTAGCCCTTTATTATAGATAATTCAGAACCAAAAAAAAGACCTAAAAATACTAAAACTAATGATATCAACTTAATATAAAAAGGTAATATAATAAAAGAAAGATCTAAGGATATAATTCATATTGTTGTACAACCAAAAAAAACTGAAAACAAGGTTAAAAAATAAATACTCATATTTATTAAAGAAAAATTTTCAAAGATAATACATAAAGGACCATGCTTATTCTTAAAAAGAACAGAATAATAAAATAACCGAATTCTATAACAACAGGTTAAGCCCAAACACAAATAAAAAATAATGAAAACGAAAAAATTAACAAAATTTATTCTAATAAGTTCTACAATTAAATCCTTTGAATAAAATCCAGAAAGAAAAGGAAATCCACATAAAGCTATAGATGAAATATTAAAACAAGAAGTAATATAAGGAATATAGTTTCTAATTAAACCTATTATACGAATATCTTGATTATTATTTATGTAATAAATCATAATACCAGAACAAAGAAATAATAAAGATTTAAACACAGCATGAGTTAATAAATGATAGAATGCTATGTTTACTATTCCTAAAAATAAAGAACTTATTATTAATCCTAATTGTCTCAAAGTAGATAAAGCAATAATTTTTTTTAAATCAAATTCATAATTAGCACAAAAAGAAGACATAAATATAGTTATTAAACTTAAAAACAATATAAATTTATTATTAAAATTAAACTGGTTAAAAAAACGAATAAGCAAATAAACCCCAGCAGTTACTAATGTAGAAGAGTGGACTAATGAAGAAACAGGGGTTGGTGCAGCTATTGCTGCAGGAAGTCAAGAAGAAAAAGGAATTTGAGCTCTTTTAGTAAAACAAGAAATAATTAATAAATAAAAAATTGACTCATGATAAAAATCTAAGTAAAATATAAAATGTCAACTCCCATAACTTATTAATCAACAAATAGAGATTAACAAACCAATATCTCCTAAACGATTTGTTAAACAAGTGATTATACCAGATAAATAACTTTTTATTGAATTATAATAAATTACCAAACAATATGAAACCATTCCTAATCCATCTCAACCTAACAAAATTCTTAACAAGTTTGGTCTAATAATTATTATTACTATACTTAAAATAAATAAAACAACCAAAAAAAGAAATCGATTTCTAGAGTAAGTATTTACCCCCATATAATCAGAACTATATAAGATTACTATAGATGAAATAAATAAAACTACTGAAATAAATATTAAAGAAATTCAATCCAACAAAATAACATAATAGACTCCAAAGGAGTTAACTCTTAAAATCTCTCACTCTAAAAATAAACAATAACCATTAAGTTGAAATAGTAGAAAAAAAAAAAAAAAAAAAAAAAAAAAGAAAAGCATAGAATAAAATCAATACAAGTAAAAATTAAATTTAAACAGATTAAAGCCTATTAAGGTTCTAGAAATCCACAATTTCTTATTTTTTATAAACTAATTCAATTAAATAAAATAATTTAAACATAATATAATAATAAATAGTGGTACTAGATGGGTAAACATTAACAAAAACTCACGAGTAAACCCTAATGAATAACAATAAAATTGATGTGGCACCCCATGCTGTATATAACTATATAAAAAATAACTGAAACATGCTGAAAAAAATGAAATAAATAAAAAGTAAAAAAAAGAAATTGATCAATATATTATTATTCTATTAATAATAAGAACTTCTCTAAAAAAATTTAGACTTGGAGGGCATCTTATATTAAAACAACAAAAAATAAATCATAAAGAAGAAACTCTAGGTATTAAATTAATTAATCCTTTGTTGATAAAAAATCTACGTCTTGTATAACGTTCATAAGAAAAATTTGCTAAACAAAATAAACCAGAAGAACAAAGACCATGAGAAATTATCATAAAGTAAGCTCCTATAATTCCCCACTTTGTTATAGAACACAATCCTAATAAACAAACACCTATGTGAGCAACGGAAGAATAAGCAATTAAACACTTAACATCAGATTGTATTAAACAAACAATCCCTACTAAAATACAACCAATAATAGACAAGGAGTATAAAATATAGCTATATAAAAAGAAAGAATATTCATATAAAGGTATAAAACGAATAAATCCATAGCCTCCAATTTTAAGTAATAACCCAGCCAAAATTATAGAACCAGAAACCGGAGCTTGAACATGAGCTTTGGGGAGCCAATAGTGAACTATGAATATTGGTAATTTAACTAAAAAAACCAAAAACATAAATATAAAAAGAATGAATCTACAAGGGATGCTATATTTTATGTCAAAGTACAAAGTTATTTCATTATTTAACAAATAGATAATAATTAAAAAAAAAGGTAATGAAGAAAACAAAGTATAAAAAAATAGGTATAAACCAGAGATTAACCGTTCAGGTTGATAACCTCAAGTATAAATTAAAATTAATAAAAAAATTAGTCTAAATTCAAAAAATAAATATATAATAAGTATATTTAATGTAGAAAAAACTAAAACTAAATTTAAACACAATATAAAACTTACAAATAAAAAAAGATTTTCTAAAAAAAAACCTTTAACAGAATTTCTAGAAATAATTATTAAAGAAATAATTACTATTCTTAAAATGATTAAACCATAAGAATAATAATCAATACCATAATTATAAGAAATACAACAGAAAAAAGGAAACTTGTTAAATATAACAAATATTAATATTAATAATAAAATTATAACTTGAAATAAATTTCAATTTATTTTTAAATAAAAAAAAGGGGTCATTATGAAAAAATATATAAATAGTTTTATCATAAAAATATACTTGATAAATAGTCATTGCCATGACAACGAATTATATAAACTAAAATTCTTAAACCTAAAACACCTTCACAAACATAAAAAGTTATTATAATTAAATATATATAAAAAATATGTCTAAATATAAGACAATATCTTATAATAATTAAAAGAAGAGTAATAATGATAAATTCTAGTCTAATTAAACATAATAAGATATGCTTACGAATTAAAACAAGACAAAATAATCCTATAAAGAATATATAAAAAAAATAAAAATTCATTTGTTTTAATAATTTAATAAAAATAATGACTTTGTAAGTCATAATTAAGTTTAAAAAGCTTTTAAAGCATCAGCAAAGTATAAAATTATACCTCTTAATTTCCCAAAAATTAAATTTTTAATAAACTATTAGCTGTTATTAAATTAATTTTAATTAAATTAATAATAATTTTGTCATCATCTACTTTATTTTTAACTAATCCTATATCAATAGGACTAATTTTAATAACACAAACTATTATGATAATTATTTATATAAACCTAATTATAACATCATCCTGATTTGCTATAGCAACATTCATAATAATAATCGGAGGATTATTAATTATATTCATATATATAAGAAGAATCGCTTCTAATGAAAAATTTAAAATTAATATCAATATTGTCCTAGTTATAGTTATTATATTAATAATTTATGATGAAATAATAATTCAACATCAAATTAATGAAAATCAAGAATTAATTTATTCAAATAATATAAATTTCTCTCTAACTAAAATTTATAATAAAAAATCAATAATAATTACAATTATGCTAGTTCTATACTTACTTTTAACAATAATTTCAGTTACCAAAATAGTTAAACATCATAAAGGACCTCTGCGAGCTATTAATTACACTTATGAATAAACCTATACGAAAAACTAATCAAATAATTAAAATTATTAATTATTCAATTATTGACCTTCCAGCTCCTGTAAATTTATCAGCTTGATGAAACTTCGGGGTATTACTTGGTATATGTCTTGTAATACAAATTTTATCAGGAATTCTACTTTCAATACACTATACTGCTGACATTCAAATAGCATTTGATAGAGTAAATCATATTACACGTAATGTTAATTATGGGTGATTAATGCGTACTTTACATTCTAATGGGGCATCTTTGTTTTTTATTTGTTTGTATATTCATACTGGTCGAGGTATTTATTATGGGTCATATAAATATTCAAAAACATGAATGTTAGGTGTTATTCTTATATTATTAACTATAGCAACAGCATTCTTGGGGTATGTATTACCATGAGGGCAAATATCATTCTGAGGAGCTACAGTTATTACTAACCTACTTTCAGCAATCCCTTATATTGGGGGTATAATTGTAAACTGATTATGAGGTGGGTTTGCAGTTGATAATGCAACCCTTTCACGTTTTTTTAGACTACACTTTTTAATACCTTTTGTTATTTTAATAATAACTGTAATTCACATTTTTTTTCTTCACACAACTGGATCTAACAACCCAATTGGTTTAAAATCTGATATTGATAAAATTCCTTTCCATCCATATTTCTCAATTAAGGATATTATAGGGGTTTGTATAATTATAATAGCACTACTAATAGTAAATTTAAGAGCCCCATATATATTATCAGACCCAGACAATTTTATTAATGCAAACCCAATAATTACACCAGTACACATTCAACCAGAATGATATTTTTTATTTGCTTATGCAATTTTACGATCAATTCCTAACAAATTAGGAGGGGTAATAGCATTATTTTTATCAATTTTAATTTTAATTATTTTACCAATTTCAATAAAAATAAAATTTAAAAGAATTTCATTTTATCCATTAAGACAATATATATACTGAATATTTGTAATAACAATAATATTATTAACTTGAATTGGGGCACGACCAGTAGAATTGCCTTACACTTTCATTGGAGTAGTACTAACAATTATATATTTTATATACTTTATTATAGACCCAATTATAGTTAAAACATGAGACAAAATAATTAATTAGATATTTAGCTTATTTTAAAGCATTTACTTTGAAAGTAAAATAAAGATTAATATTTAATATCTTTTACAAAAAAAAATGATAATAAAATATTAACTTTAATAAAACAAAGAAAATAACAAAATTTAGAGATAAAGGTAAATAACACTTTCATGCTAAATATATTAGTTTGTCATAACGATAACGTGGTAATGAACAACGAACCCAAATAAACAAAAAACAAACTAAAATAATCTTAATAAAAAATATGAAGGAATAGTAATCCCCACCTACAAAGATTATGCAAGTTAATAATCTTATAAAAATAATTCTAGCATATTCAGAAATAAATAATAAAGCAAAACCACCAGAGCCATACTCAACATTAAATCCAGAAACTAGTTCTCTTTCTCCTTCAGAAAAATCAAATGGTGATCGATTTGTTTCGGCTAATATACAAGAAAACCAACAAAAGAAAATAGGCAGTGAAAAGAAAATAAATCAACTTAAATTTTGGATTATAATAAAATTAATTATATTGTAACTATCACATAACAAAAAAAAACCTAACAAAATAATAGATAATGAAACTTCATAAGAAATAGATTGAGAGACACTTCGAATACAGCCAAGAATTGAGTATACTCTATTAGATGATCAACCACAAATGATTAAAGAATAAACACCTAACCTTGAAACACACAAGAAAAATAAAAACCCAAAATCAAAATTAATACAATTTACATAAAAAGGAAATAAAAGTCAAATAAATAAAGATTGAATAAGACCAAGACAAGGACAAATATAGTAAATTAAAAAGTTAGAATTAACAGGAAAAAATTGTTCCTTTAGAAATAACTTTTCACCATCTCTAAAAGGTTGTAATAGACCTATAAATCCAACTTTATTCGGGCCTTTTCGAATGTGTATATAACCTATAATATTTCGCTCCAATAAAGTAAAGAACCCAACAGAAACTATAATTAATGCTAATATAATTAAACTAGTAATATAATAAATTAATGAATGTGAATTAATAATCACTTAATTAAACTCTAAACTTAATACATAATCTGTCAATTCATTATTTAAAACAAAATATTTAAAATAAATTAATTGGTCCTTTCGTACTAAATTAAATTATAAACTTTAAGATAGAAACCAACCTGGCTCACACCGGTTTGAACTCAAATCATGTAAGAATTTAAAAGTCGAACAGACTTAATATAATAAATACTTCTTTATTAATTTATCTTAATTCAACATCGAGGTCGCAAACTTTTATATAGATATGAACTCCCCATAAATATTACGCTGTTATCCCTAAGGTAACTTTATCTTTTTATAAAATAAATTTAAATCAAAATAAACATAAATTAATGAAAATAGAAAATAAAGATTAATTTATTTATCACCCCAATAAAATTAAATTTATAAAATAAAGTAAATATTAAACAAAAATTTAAAAATTAAATAAAGTTCTATAGGGTCTTTTCGTCCCAAAAAAATAATTAAGCTTTTTTACTCAAAAATAAAATTAAATAATTAAATTTAATAAAATTAATTTCTCATTAAATCTTTCATTCCAGTCCTCAATTAAAAAACTAATTATTATGCTACCTTTGTACAGTTAAAATACTGCAGCCATTTAAATATTCATAGGGCAGAATTTACCTTTAATAGAATCTAAAAGAAATGTTTTTGATAAACAGTTGAAAATTAAATTTGTCGAATTCATTCAATTTCAATTTAAAAATTTACTAATATAATCAAAATTAAAATAAAAATAAAGATTTGTTAATAAATTTACTTAAGAAATAAATTATATAAAATCATTATTAAAGTATTTAAATATATTAATAACTTAAATTAAAATTTAAAAAATAATAAAAATATTTTGAATATAAATTTTATCAATAAATAAAGATTATCCCTTATTAAATACGAATTAAAATAAAAATTTGTAAATTTTATTTAATCATAAATAACCAGATATAATAAAAATCGAATAGAATATATCTACAAAAAAAAATTGTAATTATTTTTTATAACAAATAAAATTAAATTTTAATTGACCTTTTTATTTCGGGAAAAGAAAATAATTAATTAAATTAATTAACCCTGATACAAAAGGTACTACAAAATTTTTGTAAAAATATAAAAAATTACCCTTAAGGAATATTATAAAATAAATTTATTAATTAACTAAAAATTGTAAAATATAAATTTTTATAATAAAATTGTATATCAGAAAAAATATTTAATTTTCAAATTAATGTAACTAATTAGCTTTAATTTATAAGCTACATTCTGAGCTTTCTAACCTCACTTTCCAGTAAAATTACTTTGTTACGACTTATCTCAACTTATATGAGAGTGACGGGCAATATGTACATAATTTAGTATAAAATTCAATTTAAATAATTATTTAAATTTACTATTAAATTCTAAATTGATAATTTAAATATTAAAGTATAATAATTATTTAAAATTAAAGTAATCCAAATCTTCTTTCTAAAAACTGCACCTTGACCTAATATAAATTTTTAAAAAAAAGAGAAAATATATAAGAAAAACATTCCAAATTATAGAGATATACAAATAAAAAGAAAGTAGAAAATATCGTGGTTTATCAATTAGAACCAGATTCCTCTAAAAATATAAACTACCGCCAAATTCTTTGAGTTTAATAGAACTTAACTATTAATATTCAAGTTAATTATTAAATTAAAAATAATAGGGTATTTAATCCTAGTCCCAAATTAAAATTTATAAAAAATTATTAAGAACATTATTTTAATAATAAATTCCCCCTAAATTTATAAATTATAAATTCAAAAAAAAAAATTAAATAATTTAAACTTAATTTATTAAATAAAATAAATTGTATAACCGCGAATGCTGGCACAATTTTAATCCATCATAATTATATTCCTAAACAAAAATAAAAATAATTTATAACATTTATTACTGAAAATTTTAGAATTAAAAATAATTCATATAACTTTTATAATAACCTAATAAATAAAGCAAGAATAAAACTTACTGTAATAAAGTCATTAAGATATAGTATATTTTTAAATAATAATTTAACATAAAAAATTTATAATAATTATATAAATATAAATAATAAATAACATTAACTGCATATTATTCACTAAACAAATTGGGAGAAAATTTGTTAGCTAATTAATTATAGTAATGTACGTCAAATCTTATAGTATACATTGGAAAAAGATATATACTATTATTAGTGTATGCCCACTATATGGATATAAACATTAACTGCATATTATTCACTAAACAAATTGGGAGAAAATTTGTTAGCTAATTAATTATAGTAATGTACGTCAAATCTTATAGTATACATTGGAAAAAGATATATACTATTATTAGTGTATGCCCACTATATGGATATAAACATTAACTGCATATTATTCACTAAACAAATTGGGAGAAAATTTGTTAGCTAATTAATTATAGTAATGTACGTCAAATCTTATAGTATACATTGGAAAAAGATATATACTATTATTAGTGTATGCCCACTATATGGATATAAACATTAACTGCATATTATTCACTAAACAAATTGGGAGAAAATTTGTTAGCTAATTAATTATAGTAATGTACGTCAAATCTTATAGTATACATTGGAAAAAGATATATACTATTATTAGTGTATGCCCACTATATGGATATAAACATTAACTGCATATTATTCACTAAACAAATTGGGAGAAAATTTGTTAGCTAATTAATTATAGTAATGTACGTCAAATCTTATAGTATACATTGGAAAAAGATATATACTATTATTAGTGTATGCCCACTATATGGATATAAACATTAACTGCATATTATTCACTAAACAAATTGGGAGAAAATTTGTTAGCTAATTAATTATAGTAATGTACGTCAAATCTTATAGTATACATTGGAAAAAGATATATACTATTATTAGTGTATGCCCACTATATGGATATAAACATTAACTGCATATTATTCACTAAACAAATTGGGAGAAAATTTGTTAGCTAATTAATTATAGTAATGTACGTCAAATCTTATAGTATACATTGGAAAAAGATATATACTATTATTATAATATAATTAATATTAAATAATTTGATATTGATTTAAAATCCTATATTGAATTGAGTTTACATCTTTATTATTAAATCATATGAGGAAATAATATAATACATTGATAATAAATATATTATTAATAATAAAATATTTGATTGAATAATCTAAATTAATAATAAAATATATTATTAATAATAAAATATTTAATTTAAATAACTACGTTGATTCATTTTCAATATTTATCGAACAAATTAATTTAAATACTAAATTTATTATTTAATCCTTTCTTTTTTTTTTTTTAGCTCTAAAAAAAAAGAAAGGTTAATTATAATTAAATTATTTATTGATATATATATATTAAATATATTAATAATAATAAAATATTTAATATAAATAATTTTAATTAAAATTTTATATTTAATTAAATCTACCTCTTTATTAGTAGAGGTAGATTTATTGAATATATATAAATAATAAATATATTATTAATAATAAAATATTTAATATATATAAATAATAAATATATTAATAATAATAAAATATTTAATATATATATATATAAATAATAAATATATAATTAATAATAAAATATTTAATATAAATAATTTTAATTAAAATTTTATATTTAATTAAATCTACCTCTTTATTAGTAGAGGTAGATTTATTGAATATATATAAATAATAAATATATTATTAATAATAAAATATTTAATATATATAAATAATAAATATATTAATAATAATAAAATATTTAATATATATATATATAAATAATAAATATATAATTAATAATAAAATATTTAATATAAATAATTTTAATTAAAATTTTATATTTAATTAAATCTACCTCTTTATTAGTAGAGGTAGATTTATTGAATATATATAAATAATAAATATATTATTAATAATAAAATATTTAATATATATAAATAATAAATATATTAATAATAATAAAATATTTAATATATATATATATAAATAATAAATATATAATTAATAATAAAATATTTAATATAAATAATTTTAATTAAAATTTTATATTTAATTAAATCTACCTCTTTATTAGTAGAGGTAGATTTATTGAATATATATAAATAATAAATATATTATTAATAATAAAATATTTAATATATATAAATAATAAATATATTAATAATAATAAAATATTTAATATATATATATATAAATAATAAATATATAATTAATAATAAAATATTTAATATAAATAATTTTAATTAAAATTTTATATTTAATTAAATCTACCTCTTTATTAGTAGAGGTAGATTTATTGAATATATATAAATAATAAATATATTATTAATAATAAAATATTTAATATATATAAATAATAAATATATTAATAATAATAAAATATTTAATATATATATATATAAATAATAAATATATAATTAATAATAAAATATTTAATATAAATAATTTTAATTAAAATTTTATATTTAATTAAATCTACCTCTTTATTAGTAGAGGTAGATTTATTGAATATATATAAATAATAAATATATTATTAATAATAAAATATTTAATATAATAATAAATATAATAATAATTTTATATAAATTAATTTTTTTGCTGTATTTTTTTTTTTATAAAATTATGTGT
